# Smartphone apps for mental health: systematic review of the literature and five recommendations for clinical translation

**DOI:** 10.1136/bmjopen-2024-093932

**Published:** 2025-02-11

**Authors:** Aljawharah Almuqrin, Ryan Hammoud, Ilham Terbagou, Stefania Tognin, Andrea Mechelli

**Affiliations:** 1 Department of Health Sciences, Princess Nourah bint Abdulrahman University, Riyadh, Saudi Arabia; 2 Department of Psychosis Studies, Institute of Psychiatry, Psychology and Neuroscience, King’s College London, London, UK

**Keywords:** Depression & mood disorders, Schizophrenia & psychotic disorders, PSYCHIATRY, MENTAL HEALTH

## Abstract

**Abstract:**

**Objectives:**

Providing adequate access to mental health services is a global challenge. Smartphone apps offer a potentially cost-effective, available and accessible solution for monitoring, supporting and treating mental health conditions. This systematic review describes and evaluates the usage of smartphone apps across a wide range of mental health disorders in terms of clinical effectiveness, feasibility and acceptability.

**Design:**

This is a systematic review of studies examining treatment, self-monitoring and multipurpose smartphone apps for mental health disorders.

**Data sources:**

Studies were identified through a comprehensive search of the Ovid and PubMed databases. Articles published up to 14 January 2024 were included based on predefined criteria.

**Eligibility criteria:**

We included randomised controlled trials that compared mental health apps (single- or multipurpose) with treatment-as-usual or no treatment for clinical populations with mental health disorders. Studies were excluded if they focused on web-based interventions, combined apps with non-TAU treatments or targeted physical health apps.

**Data extraction and synthesis:**

Two independent reviewers screened and selected studies, with a third reviewer resolving inconsistencies. Extracted data included study details, participant characteristics, app information and outcome measures related to effectiveness, feasibility and acceptability. A risk-of-bias assessment for each study was conducted.

**Results:**

Out of 4153 non-duplicate articles screened, 31 studies met full-text eligibility criteria. These included 6 studies on treatment apps, 4 on self-monitoring apps and 21 on multipurpose apps for a range of mental health disorders. Fifteen were identified as having between some and high concern on the risk-of-bias assessment. While smartphone apps were generally effective and acceptable, their feasibility appeared to decline over time.

**Conclusions:**

Smartphone apps are promising tools for mental healthcare, demonstrating effectiveness and acceptability. However, challenges such as reduced feasibility over time, potential biases and underrepresented demographics require further research. This review proposes five recommendations for improving clinical translation in future studies.

**STRENGTHS AND LIMITATIONS OF THIS STUDY**

- The review systematically evaluated smartphone apps for mental health using a comprehensive search strategy and robust risk-of-bias assessment.
- The studies were included from diverse clinical contexts, distinguishing between clinical effectiveness, feasibility and acceptability.
- The included studies were limited by a relatively homogeneous sample population, primarily middle-aged women, with reduced representation of adolescents and older adults.
- Many studies (15 of 31) raised concerns regarding the risk of bias, potentially limiting the reliability of the findings.
- No studies addressed the use of smartphones for obsessive-compulsive disorder, highlighting a gap in app-based mental health interventions.

## Introduction

 Approximately 1 billion people worldwide are affected by mental disorders, posing a global challenge.^1^ The WHO estimates that 50% of people with mental disorders lack access to care in developed countries, and this percentage increases to 85% in the developing world.^2^ One potential solution is through the use of smartphone-based mental health apps, which can provide support for individuals in need. Currently, there are 6.3 billion smartphone users globally, with over 90% using apps daily.^3^ A recent survey found that 71% of psychiatric patients wanted to use apps to supplement their clinical care.^4^ Therefore, it is no surprise that apps have gained substantial interest in healthcare settings, with currently over 20 000 mental health apps available on the market.^5^

There are three types of mental health apps: treatment, self-monitoring and predictive.^6 7^ Treatment apps provide a variety of psychological interventions, such as those based on cognitive-behavioural therapy. They have been shown to reduce symptoms like depression and anxiety,^8–10^ and enhance psychiatric patients’ quality of life^8^ and their recovery.^9^ They can be used in conjunction with other therapeutic approaches or independently, particularly for managing milder cases or supporting users until they can access specialised care. Self-monitoring apps allow patients to track changes in their mood and symptoms, which increases their emotional self-awareness (ESA).^11^ Increasing ESA has a positive effect on psychiatric patients as it improves their coping skills and decreases the severity of their symptoms.^12 13^ Finally, predictive apps monitor and predict clinical relapse, allowing for early intervention through preventing and stabilising symptoms.^14^ Additional features of mental health apps include improving healthcare efficiency,^15^ psychoeducation, clinical assessment, skills training, tracking treatment progress and communication with healthcare professionals.^16^

Using mental health apps offers several potential advantages. First, mental health apps are cost-effective^17^ since they directly reduce hospital admission costs.^18^ Second, mental health apps are often readily available and accessible, unlike conventional in-person interventions.^19^ Third, mental health apps provide access to an extensive population, including those who live in rural areas with limited access to mental health services.^20^ Fourth, mental health apps lead to higher engagement with mental health services. Some people may prefer to communicate with mental health professionals via smartphones rather than in person. It is especially well-suited for participants aged from 14 to 24 years, who are usually most affected by mental health issues and least likely to seek help, as mobile phones are their preferred mode of communication.^21^

Despite the increasing number of mobile apps for mental health, actual usage rates and perceived usefulness remain relatively low. A recent systematic review found that while approximately 87% of individuals with mental disorders owned smartphones, only 23% used them for mental health purposes, suggesting significant barriers to uptake and usage.^22^ Similarly, Kim et al^23^ (2022) highlighted that while mobile apps can reduce symptoms of severe mental illness, challenges such as user engagement and operational complexity hinder their integration into clinical care. These barriers underline the importance of systematically assessing the clinical effectiveness, feasibility and acceptability of mental health apps to support their broader adoption in mental healthcare settings.^24–26^ Clinical effectiveness assesses app efficacy compared with treatment-as-usual (TAU).^27 28^ While previous reviews emphasised that mental health apps are effective in terms of improving functioning and quality of life and reducing symptoms,^29–33^ many included biased studies, leading to inconclusive results.^34 35^ Therefore, further systematic reviews on the clinical effectiveness of mental health apps are needed.

Feasibility is an objective that measures usage and retention rates among the patients,^36^ a crucial measure as mental health services prioritise apps with proven feasibility.^37^ A systematic review comparing seven studies demonstrated that mental health apps have high feasibility (92% retention rate, 72% response to prompts and 3.95 interactions with the app per day),^38^ but only for a narrow range of mental health disorders. This highlights the need to assess the feasibility of apps related to a larger range of mental health disorders.

Acceptability is a subjective measure of patient usage and satisfaction.^39^ Prior studies frequently interchanged the terms ‘acceptability’ and ‘feasibility’,^40^ resulting in unclear findings. A systematic review comparing eight studies emphasised that using mental health apps is highly feasible.^41^ However, it did not clearly define feasibility, often mixing it with acceptability. Further research is needed to clearly differentiate between these concepts.

### Aims and objectives

This systematic review assesses clinical effectiveness (primary outcome), feasibility and acceptability (secondary outcomes) of mental health apps compared with TAU. We address the following research questions: (1) to what extent are current mental health apps clinically effective?, (2) what is the feasibility of using mental health apps? and (3) what is the acceptability of mental health apps?

## Methods

### Protocol

This systematic review followed the Preferred Reporting Items for Systematic Reviews and Meta-Analyses (PRISMA) guidelines,^42^ and the protocol was registered to the international Prospective Register of Systematic Reviews (CRD42020193699).

### Patient and public involvement

Patients and/or the public were not involved in the design, conduct, reporting or dissemination plans of this research.

### Inclusion and exclusion criteria

The inclusion criteria were as follows: (1) randomised controlled trials (RCTs) reporting on a primary intervention using a mental health app (single- or multipurpose app) compared with TAU or no treatment; (2) articles reporting on clinical samples from inpatient or community settings with various mental health disorders such as depression, anxiety, phobia, panic disorder, obsessive-compulsive disorder, post-traumatic stress disorder (PTSD), psychosis, bipolar disorder, suicidal ideation/behaviour and self-harm; (3) original articles in peer-reviewed journals and (4) articles published in English.

The exclusion criteria were as follows: (1) articles reporting on web-based interventions not requiring apps; (2) articles that used mental health apps in addition to interventions other than TAU; (3) articles on apps with a focus on physical health; (4) observational studies.

### Information sources and search strategy

This systematic review conducted a comprehensive search that started in June 2020 and the final search was conducted on 14 January 2024, using PubMed and Ovid databases (composed of APA PsycInfo, Global Health, Embase and Ovid MEDLINE). The searches were run four times during this period to ensure that the results remained up to date as the review progressed and to incorporate newly published studies relevant to the topic. Search terms related to (1) mental health, (2) smartphones and (3) self-management were used. Appendix A provides an example of the full search strategy, including applied limits for one of the searches (eg, RCTs), while other searches used no additional filters.

### Selection and data collection process

Two reviewers independently conducted the search and screened articles based on the inclusion and exclusion criteria with a third reviewer resolving inconsistencies. This process ensured the reliability and consistency of study selection. Extracted data included article details (authors and publication year), participant information (sample size, gender, mean age, inclusion/exclusion criteria, diagnosis and diagnostic tool), mental health app information (name and type) and outcome measures (clinical effectiveness, feasibility and acceptability).

### Data items

The primary outcome of this review, clinical effectiveness, is defined as the extent to which an app effectively achieves its intended purpose.^27^ For self-monitoring apps, this was assessed by assessing the effect of TAU compared with those who are also using self-monitoring apps. For treatment and prediction apps, clinical effectiveness was represented by intention-to-treat analysis or analysis of covariance.

Secondary outcomes of this review are with regard to feasibility and acceptability of mental health apps. Feasibility is defined as an objective measure indicating the ease of psychological intervention.^36^ The feasibility was measured by overall usage and retention/attrition rates. Acceptability is defined as a subjective measure of psychiatric patients’ attitudes towards mental health app usage^39^ and was assessed through the use of satisfaction questionnaires.

### Risk-of-bias assessment

This systematic review assessed errors and bias in the article selection process. For example, randomisation such as blinding degree, allocation and attrition were determined by the reviewer. In addition, to assess the risk of bias in the article selection process, this systematic review used the revised Cochrane risk-of-bias tool (RoB 2) for randomised trials.^43^

### Synthesis of results

A narrative synthesis was conducted for the outcomes (ie, the clinical effectiveness, feasibility and acceptability of mental health apps). This narrative synthesis consisted of all eligible articles that met the inclusion criteria and showed a comparison between mental health apps and TAU in their effectiveness in self-monitoring, treatment and predicting.

## Results

### Study selection and characteristics

**Figure 1:**
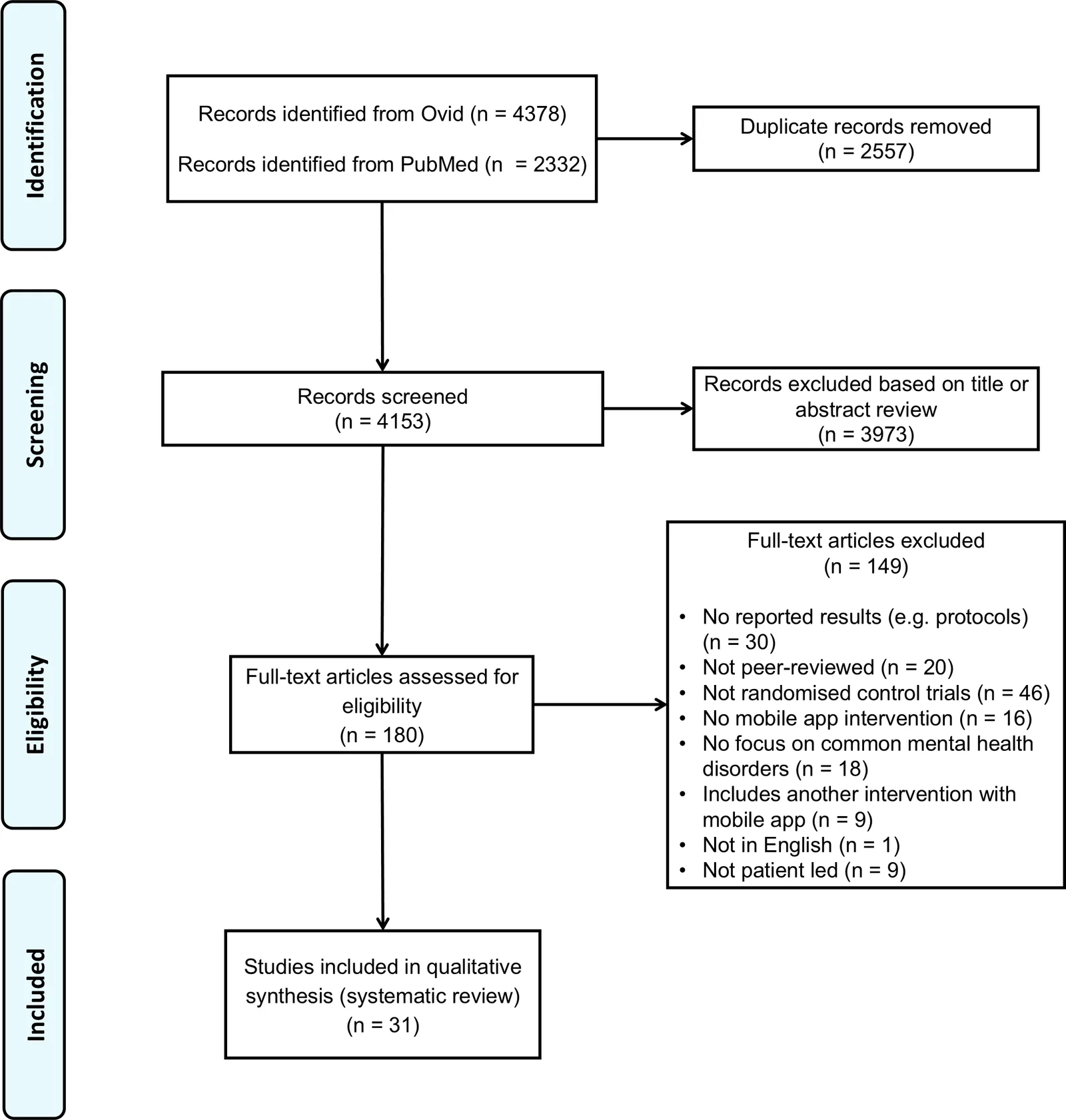
PRISMA flow diagram. PRISMA, Preferred Reporting Items for Systematic Reviews and Meta-Analyses.

Figure 1 shows the PRISMA flow chart of the search results. Of note, 31 articles reporting on 27 different mental health apps were identified. These articles are summarised in online supplemental table 1. Six articles discuss treatment applications, 4 articles discuss self-monitoring applications and the remaining 21 articles discuss multipurpose applications, including a combination of tracking, self-monitoring and/or treatment components.

This systematic review consisted of a total of 3660 participants with a mean age of 28.29 years. Most studies specified eligibility criteria as participants older than 18 years and younger than 60 years.

Notably, 24 of 30 articles had more than 50% female participants. In addition, seven articles had more male participants due to the population of interest (ie, veterans in Possemato *et al*^44^). Further details on the location of each study and duration of app usage can be found in online supplemental table 3.

### Risk of bias

**Figure 2:**
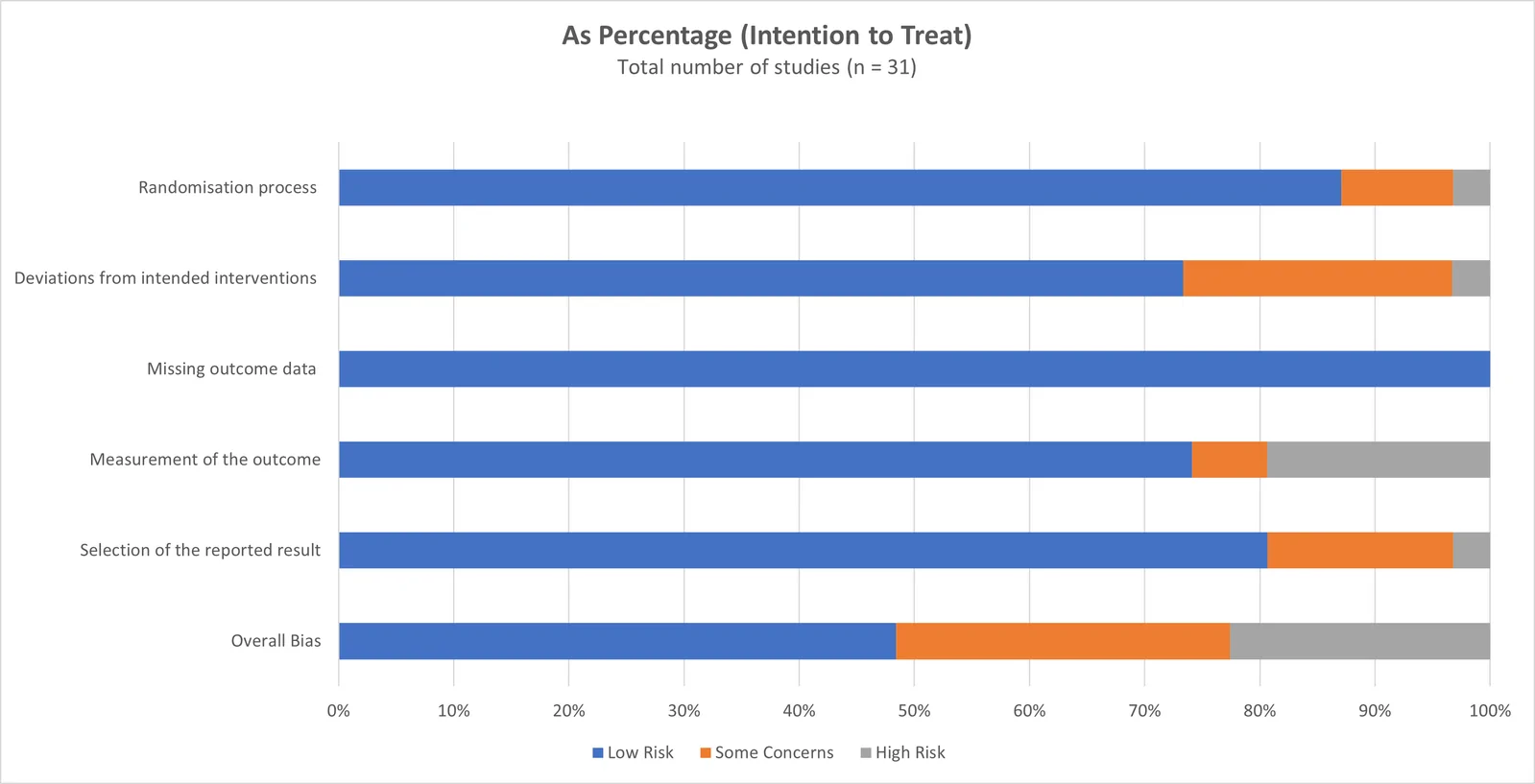
Overall risk of bias.

This systematic review used RoB 2 to assess the risk of bias for included RCTs.^43^ Overall, 48.4% of RCTs had a low risk of bias, 29% raised some concerns and 22.6% had a high risk of bias. Figure 2 shows a high risk of bias in the outcome measures (19.4 %), while missing outcome data, the randomisation process and the selection of reported results showed lower bias (100%, 87.1% and 80.6%, respectively).

**Figure 3:**
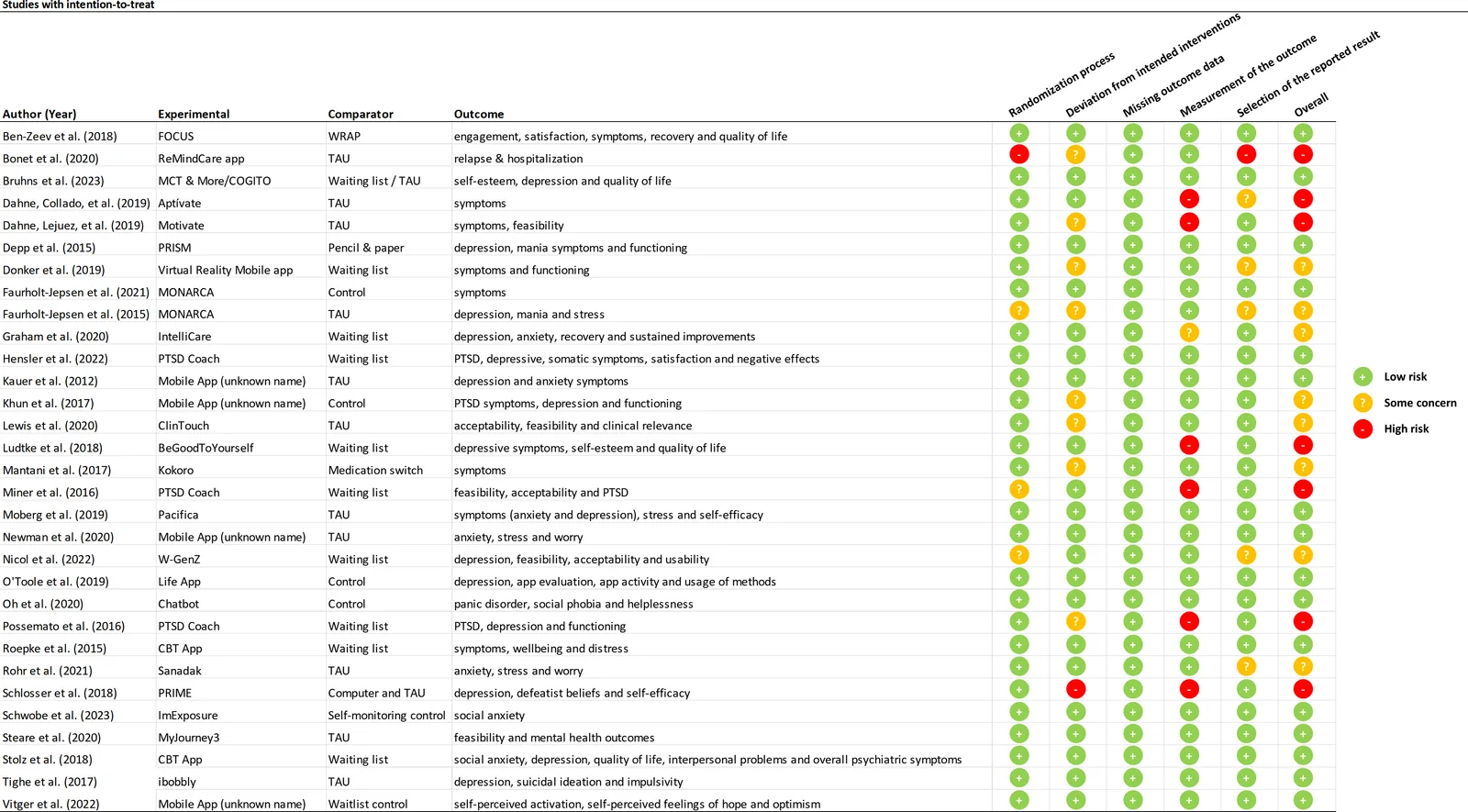
Individual risk of bias.

The risk of bias for each RCT is described in-depth in figure 3. Most RCTs presented low bias from the randomisation process, with the exception of Miner *et al*,^45^ which lacked information about participant concealment, potentially affecting motivation and adherence in the control group.

Six RCTs raised concerns in the selection of reported results,^46–51^ possibly due to multiple analyses to assess changes in symptoms^48^ or not pre-specifying their data analysis.^47^ Seven RCTs reported a high risk for bias for outcome measures,^44 45 47 52–54^ often due to unblinded assessors^47 52 54^ or insufficient information.^44 45 53^

### Key findings

This systematic review assessed the clinical effectiveness (primary outcome), feasibility and acceptability (secondary outcomes) of mental health apps. Outcomes are assessed using data from treatment, self-monitoring and multipurpose mental health apps, as no article assesses single-purpose predictive applications. The findings are shown in online supplemental table 2.

### Effectiveness, feasibility and acceptability of treatment apps

This systematic review assessed six studies on the treatment mental health apps in terms of effectiveness, acceptability and feasibility. Four studies demonstrated a statistically significant effect, reducing symptoms such as acrophobia,^48^ depression^55^ and anxiety symptoms.^56 57^ Notably, several studies also reported improvements in quality-of-life metrics for patients.^48 55–57^ However, Röhr *et al*^50^ found no impact on PTSD symptoms but significantly lowered self-stigma.

In terms of feasibility, Stolz *et al*^56^ found interaction levels with apps compared with personal computers (d=0.14, p=0.01), suggesting greater feasibility. Similarly, Röhr *et al*^50^ reported low dropout rates (12.8%), but Donker *et al*^48^ and Roepke *et al*,^55^ found lower retention rates at post-test (59% and 26.15%, respectively) and follow-up (49% and 18.34%, respectively).

In terms of acceptability, Lüdtke *et al*^53^ found treatment apps acceptable, with over 50% positive responses on the Client Satisfaction Questionnaire (ZUF-8; Schmidt *et al*^58^), consistent with Donker *et al*.’s^48^ user-friendliness scale^59^ results. While Donker *et al*^48^ reported a ‘good’ score on the user-friendliness scale, indicating overall user satisfaction and acceptability, there remains potential for further improvement to enhance user satisfaction.

### Effectiveness, feasibility and acceptability of self-monitoring apps

This systematic review assessed three studies on self-monitoring mental health apps in terms of effectiveness, acceptability and feasibility. Bonet *et al*^46^ found that using a self-monitoring app is effective, reducing hospitalisations (χ^2^=4.6, p=0.03), relapses (χ2=13.7, p=0.001) and urgent care visits (χ2=7.4, p=0.006),^46^ though Steare *et al*^60^ found no significant impact on clinical outcomes, noting that the trial was not statistically powered to detect effectiveness, and Lewis *et al*^61^ reported effectiveness primarily in early psychosis, with limited benefits for chronic illness.

Bonet *et al*^46^ and Steare *et al*^60^ reported compliance rates between 85% and 100%, suggesting strong engagements with their respective apps. In Lewis *et al*,^61^ feasibility was reflected by a 95% retention rate over 12 weeks, with 84% of participants achieving acceptable adherence.

Lewis *et al*^61^ demonstrated high acceptability, with 84% of participants responding to at least 33% of alerts, indicating regular app usage. Similarly, qualitative feedback from Steare *et al*^60^ suggested that many participants found the app acceptable and reported clear benefits. However, Steare *et al*^60^ noted that participants expressed concerns about data privacy and lack of clinician support, which may have impacted long-term engagement. Bonet et al^46^ (2020) also observed lower acceptability for participants who were suspicious of technology (33%) or found the app boring and not beneficial (40%).

### Effectiveness, feasibility and acceptability of multipurpose apps

Twenty-two studies investigated the use of multipurpose apps combining treatment components, self-monitoring or prediction components. Sixteen of these studies demonstrated treatment component effectiveness compared with control conditions.^45 47 49 51 54 62–72^ For example, Ben-Zeev *et al*^73^ showed improvements in quality of life (t=2.55, p=0.001), and Graham *et al*^63^ reported recovery odds for depression (OR: 3.24, 95% CI: 1.54, 6.86) and anxiety (OR: 2.17, 95% CI: 1.08, 4.36). However, Possemato *et al*^44^ and Bruhns *et al*^74^ found that treatment apps had no significant impact compared with other interventions. Self-monitoring component effectiveness was shown in three studies^52 54 65^ and the prediction component effectiveness in one study.^75^

Feasibility of multipurpose apps was assessed in 14 studies, with 11 finding high compliance, retention and usage rates. For example, Dahne *et al*^52^ reported retention rates of 90% in the first week and 50% in 8 weeks, and usage rates of 71%. While Depp *et al*^62^ reported retention rates of 93% after 12 weeks, they indicated that retention had dropped by week 24. Dahne *et al*^47^ and Graham *et al*^63^ reported high retention (81% and 72.2%) and usage rates (81.8%). These findings were supported by eight other studies.^45 51 54 64 68 72 73 76^ However, Possemato *et al*^44^ found higher retention with clinical support, while Moberg *et al*^66^ reported increased attrition rates in individuals with app access.

Acceptability of multipurpose apps was assessed in nine studies, with Depp *et al*^62^ reporting a higher acceptability rate (9/10) compared with controls (8/10). This was supported by several other studies,^44 51 68 70 71 73 74^ with Miner *et al*^45^ reporting greater convenience for self-monitoring symptoms compared with traditional methods.

## Discussion

The present systematic review assessed the use of smartphone-based mental health apps for common mental health disorders focusing on clinical effectiveness, feasibility and acceptability. We identified 31 articles reporting on 27 mental health apps for treatment, self-monitoring and multiple clinical purposes. To the best of our knowledge, this is the first review evaluating these aspects across a wide range of mental health disorders with a rigid risk-of-bias assessment.

### To what extent are current mental health apps clinically effective?

The clinical effectiveness of mental health apps, defined as the effectiveness of the app compared with TAU,^28^ was assessed for treatment, self-monitoring and multipurpose apps. Four of six studies found that treatment apps reduced symptoms and improved functioning and quality of life of psychiatric patients.^48 55–57^ Two studies found no significant effect on symptoms; however, PTSD self-stigma was reduced,^50^ and symptoms improved over time.^53^ However, biases affected results, and low-bias studies were inconclusive, showing significant improvements only with clinical support.^34 35^ Further research into the effectiveness of treatment apps is required.

Self-monitoring apps showed mixed results. Only one app led to fewer hospitalisations, relapses and urgent care visits,^46^ but it had a high risk of bias. The finding was inconsistent with those of Steare *et al*^60^ and Lewis *et al*^61^ who found no significant differences between groups. Thus, further development and validation are required.

Multipurpose apps were generally effective, but not all individual components were assessed separately. Treatment components were individually effective in some studies,^45 47 49 51 54 62–72^ but the inclusion of clinical support led to better outcomes.^44^ Self-monitoring components were assessed by three studies, all of which found that they were clinically effective.^52 54 65^ A predictive feature showed effectiveness in one study.^75^ However, as this was just a single study, more research is needed to make assertive conclusions.

Overall, most apps were clinically effective, but biases and the small number of studies suggest that further research is necessary.

### Is it feasible to use mental health apps?

The feasibility of using mental health apps, defined by an objective measure of usage and retention rates,^36^ was high for some treatment apps, with higher interaction levels than personal computers^56^ and low dropout rates in certain studies.^50^ However, attrition remains a common challenge for mental health apps. For example, Roepke *et al*^55^ reported retention rates as low as 26.15% at post-test and 18.34% during a 6-week follow-up, highlighting the difficulty in sustaining engagement over time. Similarly, Dahne *et al*^52^ reported retention rates of 90% in the first week and 50% in 8 weeks. This decline in feasibility may be due to poor user engagement, repetitive tasks and privacy concerns. Torous *et al*^77^ noted that many mental health apps suffer from poor usability and lack of user-centric design. Such issues can make apps difficult or unenjoyable to use, leading to a loss of interest over time. Aryana *et al*^78^ emphasised the importance of designing apps that adapt to diverse user contexts and involve user feedback during development. This suggests that while short-term feasibility is promising, long-term retention requires further exploration, potentially through strategies such as enhanced app design and clinical support. More studies are needed to assess long-term feasibility.

Three studies included in this review found high compliance rates of self-monitoring apps,^46 60 61^ with Lewis *et al*^61^ also finding high compliance rates with clinicians. However, as mental health services prioritise the deployment of feasible apps,^37^ more studies exploring feasibility of self-monitoring apps may be required.

Fourteen studies on multipurpose apps found high in compliance, usability and retention.^45 47 51 52 54 56 62–64 68 72 73 76^ Possemato *et al*^44^ found higher retention with clinical support, and Moberg *et al*^66^ reported increased attrition. It is noteworthy that the findings presented here likely depend on the overall study period and specific app features, also relating to user acceptability.

### What is the acceptability of mental health apps?

Acceptability, measured by patient usage and satisfaction with mental health apps,^39^ was high for treatment apps.^48 53^ Self-monitoring apps were generally acceptable,^60 61^ but Bonet *et al*^46^ found them less acceptable for participants who suffer from delusions.

Nine studies assessing multipurpose apps found high acceptability, with patients finding them easy to use, convenient and helpful.^44 45 51 68 70 71 73 74^ Possemato *et al*^44^ noted improved satisfaction in those with clinical support. However, differences in app design, target populations and clinical contexts may influence overall acceptability, highlighting the importance of tailoring apps to user needs.^77 78^

In summary, acceptability ratings were high, but evidence suggests that they could improve with clinical support, indicating apps might be best used alongside TAU. Bonet *et al*^46^ found acceptability varies by target population, being less suitable for some disorders than others (such as those with delusions or paranoia). The small number of studies makes it challenging to analyse by disorder, highlighting a need for further research.

### Limitations of current smartphone applications

Despite the many benefits, mental health apps have limitations. First, while it is important to note that a majority of the global population uses smartphones,^3^ most users come from higher-income households,^79^ limiting access for those with lower socioeconomic status. While mental health apps can improve accessibility, particularly for individuals in areas with limited mental health services, their effectiveness may be hindered by barriers such as unreliable internet connectivity in rural regions, which can restrict their functionality and impact.^80^ Furthermore, digital literacy and the risk of digital exclusion present significant challenges, especially in individuals who are still unfamiliar with smartphones or apps.^81^

Second, some mental health applications are only available for either Android or iOS smartphone operating systems (eg, Dahne *et al*^47^ ^52^), highlighting the importance of multiplatform development for inclusivity.

Third, a lack of integration with clinical practice is another issue, as data from apps are often not incorporated into electronic health records. These data would be beneficial for clinicians to monitor their patients’ conditions^52^ and better understand the disorders, allowing for a more holistic approach to care for psychiatric patients.

Fourth, data and privacy concerns present a significant challenge.^82 83^ Some patients expressed wariness about confidentiality,^55 66 76^ and in some instances, they were uncomfortable responding to self-assessments in a public setting.^55^ Additionally, data breaches or unauthorised access to sensitive health information could significantly erode trust in mental health apps, a significant barrier to user engagement.^77^ These concerns can be mitigated by providing transparent privacy policies, adhering to robust encryption standards and complying with regional data protection laws. Additionally, offering the option to complete assessments at a time when they are in a private setting^45 76^ can further address privacy concerns. Addressing these concerns is critical for safeguarding user trust, ensuring confidence and supporting sustained app usage.

Finally, a limitation of smartphone applications is missing data due to patient disinterest or lack of engagement. Schlosser *et al*^54^ found self-monitoring features were the least popular, seen as repetitive and tiresome. This can be mitigated by collecting passive data and enhancing app design to increase adherence and engagement.

### Strengths and weaknesses of the current systematic review

Our findings add to the growing literature on digital technologies for mental health distinguishing between clinical effectiveness, acceptability and feasibility, and using a robust risk-of-bias assessment.^60^

However, there are several limitations in the current review. First, the sample was relatively homogeneous, mostly middle-aged female participants. Only Kauer *et al*^75^ included adolescents, and no study included a sample with a mean age of above 50 years. This underrepresentation of older adults is particularly notable, given the barriers associated with this demographic. Older adults may face additional challenges, such as lower digital literacy and unfamiliarity with smartphone apps,^84^ which could impact the feasibility and acceptability of these interventions for these groups. Thus, the findings cannot be generalised to a wider population, highlighting an understudied group in digital technologies literature. Future research should prioritise the inclusion of older adults to ensure that mental health apps are developed and validated for diverse age groups.

Furthermore, the geographic distribution of the studies (online supplemental table 3) also limits the generalisability of the findings. Notably, 14 of the 31 studies were conducted in the United States, with relatively few studies from low- and middle-income countries. This geographic bias means that the findings may not be fully applicable to populations in developing countries, where mental health apps could be crucial due to a lack of mental health services, resources and access to care. This underscores the need for further research that includes a greater diversity of locations, particularly in regions where digital health interventions could have a significant impact.

Second, the studies included are of varying quality, with 15 out of 31 studies showing some to high concern in the RoB 2 assessment. The varying levels of bias have important implications for interpreting the findings, as high or unclear risks of bias may overestimate the effectiveness, feasibility or acceptability of mental health apps. For example, while some high-risk studies, such as Bonet *et al*,^46^ reported high effectiveness with reductions in hospitalisations and relapses, these must be interpreted with caution due to methodological limitations. Issues such as inadequate randomisation, lack of blinding and selective reporting were observed in several studies, limiting the robustness of their results and emphasising the need for standardised measures. Despite the potential for low-accuracy studies to yield positive results,^34 35^ the current review prioritised studies with low risk of bias in its conclusions. These limitations highlight the need for future research to adopt robust study designs and transparent reporting to strengthen the evidence base for mental health apps.

Third, this review did not include single-purpose predict applications due to a lack of such studies. However, one study incorporated a predictive feature within a multipurpose app, demonstrating clinical effectiveness through improved ESA and reductions in depressive symptoms.^75^ This finding highlights the potential of predictive applications in mental health and suggests a need for more focused research to explore their effectiveness, acceptability and feasibility within digital health interventions.

Finally, the review excluded certain mental health conditions, such as substance use disorders and neurodevelopmental disorders, as these were outside the scope of the current review which focused on common mental health disorders. As a result, the findings may not be generalisable to these populations. Additionally, no articles were found comparing smartphone applications with TAU for OCD, indicating a need for app development targeting OCD symptoms.

### Five recommendations for clinical translations

In this final section, we propose five recommendations that should be implemented in future apps to assist with the treatment and monitoring of mental health disorders.

1. **Apps should be developed using a multiplatform framework to widen compatibility with a variety of devices:** existing tools often support either Android or iOS operating system. Considering the relatively even distribution of iOS and Android operating systems in certain markets (eg, 50.5% iOS and 48.9% Android in the United Kingdom in March 2022; https://gs.statcounter.com/os-market-share/mobile/united-kingdom/#monthly-201112-202112, accessed April 2022), future smartphone applications should support both platforms to be inclusive of the psychiatric population as a whole.
2. **Apps should provide feedback to clinicians and patients**: existing tools often lack integration with patient electronic health records and predictive features. Feedback of data and predictive information to clinicians and patients would allow for a more responsive and effective treatment approach. Apps could also allow clinicians and patients to interact, for example, via messaging services, to allow clinicians to use the information during sessions.
3. **Privacy and data protection should be a core value of the app:** several studies highlighted patients’ concerns over the confidentiality of their data.^55 66 76^ Robust encryption and authentication methods to ensure patient confidentially should be implemented during the development of the app, with frequent security audits to maintain trust. Developers should provide transparent, accessible privacy policies and data use statements. All data should be stored in accordance with data protection laws and guidelines.
4. **User experience of apps should be taken into careful consideration:** in previous studies, patients often reported disinterest when using mental health applications which resulted in missing data and decreased usage.^46^ User experience should be an important value during the development of smartphone applications to maximise feasibility and accessibility. Focusing on usability, reducing repetitive tasks incorporating a user-centric design can improve feasibility.^77^ Focusing on passive data collection and investing in the design of an app can increase its appeal and support long-term retention. Additionally, involving end-users, particularly those from diverse demographic groups who may face challenges with digital literacy, can ensure apps are tailored to meet varying needs.
5. **Involve people with lived experience of mental illness during development and validation:** while the current systematic review identified 27 studies evaluating the effectiveness, feasibility and acceptability of existing mental health apps, previous studies have reported the benefits of involving service users during the development of mental health-based apps.^34 85 86^ Therefore, future mental health apps should involve service users early in the development stage.

## Conclusion

A key aim of using smartphone apps for mental health is to provide tools that can monitor, support treatment and predict future clinical outcomes. This review found a limited number of validated smartphone apps that have been assessed in terms of clinical effectiveness, feasibility and acceptability. Overall, smartphone apps are effective and acceptable tools, though feasibility may vary over time, and some studies show bias concerns. As the usage of digital technologies in several fields is quickly evolving, improving effectiveness, feasibility and acceptability is crucial. Future studies should focus on identifying and addressing barriers to long-term feasibility, while emphasising the inclusion of diverse populations. Despite these limitations, smartphone apps offer a cost-effective means to expand resource availability and improve access to care. We hope that this review will assist with the future development of effective, feasible and acceptable smartphone apps for mental health disorders.

## Supplementary material

10.1136/bmjopen-2024-093932 online supplemental file 1

## Data Availability

Data sharing not applicable as no datasets generated and/or analysed for this study.
