# Supplementary material for: Smartphone apps for mental health: systematic review of the literature and five recommendations for clinical translation
Source: BMJ Open. 2025 Feb 11;15(2):e093932. doi: 10.1136/bmjopen-2024-093932 (PMC11815452; doi:10.1136/bmjopen-2024-093932)
Supplement: online supplemental file 1 [file bmjopen-15-2-s001.docx]

**Supplementary Table 1.** Study Characteristics

| Authors (Publication year) | N | Female % (Male %) | Mean age | Inclusion criteria | Exclusion criteria | Diagnosis | Diagnostic tool | Mobile application name | Type of mobile application |
| --- | --- | --- | --- | --- | --- | --- | --- | --- | --- |
| Ben-Zeev et al. (2018) | 163 | 41%  (59%) | 49 | Diagnosed with schizophrenia, schizoaffective disorder, bipolar disorder, major depressive disorder, age 18+, RAS^15^ (>3) | Hearing, vision, or motor impairment, less than grade 5 English reading ability and exposed to WRAP or FOCUS before | Transdiagnostic | Chart diagnosis | FOCUS | Multipurpose application (treatment & tracking) |
| Bonet et al. (2020) | 90 | 27%  (73%) | 32.8 | Diagnosis according to the DSM-5, 17- 65 years old, smartphone ownership with an internet connection, less than 5 years of illness duration | Lack of ability to use mobile device and the internet, refusal to sign an informed consent form, level of Spanish or English not fluent | Psychosis | DSM-5^6^ | ReMindCare App | Self-monitoring application |
| Bruhns et al. (2023) | 159 | 55.4%  (44%) | 39.04 | Age 18+, diagnosis of depression according to ICD-10 and DSM-5, pending discharge after day care/inpatient informed consent, internet access and possession of a smartphone, willingness to participate all aspects of study | If inclusion criteria were not met | Depression | DSM-5^6^ and ICD-10 | MCT & More/ COGITO | Multipurpose application (treatment & tracking) |
| Dahne, Collado, et al. (2019) | 42 | 67%  (33%) | 36 | Age 18+,2 own smartphones, willingness to use a phone for examination purposes and be treated through phone check email at least once a day Spanish language preferences and fluency PHQ^5-^8 (>10) seen by a doctor in last year | Scoring BDI-2^14^ (<13) psychotherapy, visually impaired endorse in suicidality | Depression | PHQ^5-^8 | Aptívate! (BA^18^) | Multipurpose application (treatment & tracking) |
| Dahne, Lejuez, et al. (2019) | 52 | 85%  (15%) | 44 | Age 18-65, willingness to use a phone for examination purposes, check email at least once a day, PHQ^5-^8 (<10) | Scoring BDI-2^14^ (<13) and current or past month indication of suicidal ideation | Depression | PHQ^5-^8 | Moodivate (BA^18^) | Multipurpose application (treatment & tracking) |
| Depp et al. (2015) | 82 | 63%  (37%) | 48 | Age 18+, outpatients and currently prescribed medications for bipolar disorder, no manual or visual disabilities | Substance use disorder hospitalized severe range for either depressive symptoms (>32) or manic symptoms (>20) and severe psychopathology | Bipolar Disorder | MADRS^2^  and YMRS^3^ | PRISM | Multipurpose application (treatment & tracking) |
| Donker et al. (2019) | 193 | 67%  (33%) | 41 | Age 18-65, scoring 45+ (AQ^1^), Android smartphone | Insufficient Dutch language skills, receiving treatment/medication, having severe depression, suicidality | Acrophobia | AQ^1^ | ZeroPhobia | Treatment mobile application |
| Faurholt-Jepsen et al. (2021) | 67 | 67%  (33%) | 29 | BD diagnosis, 18-60 years old, HDRS-17 ≤17 and YMRS score ≤ 17 | Pregnancy, a lack of Danish language skills, inability to learn the technicalities for using a smartphone, unwilling to use the trial smartphone as the primary cell phone, and severely physical illness or schizophrenia, schizotypal or delusional disorders according to the SCAN interview | Bipolar Disorder | ICD-10 and DSM-IV using SCAN interview | MONARCA | Multipurpose application (treatment & tracking) |
| Faurholt-Jepsen et al. (2015) | 78 | 67%  (33%) | 29 | Bipolar (ICD-10^13^), age18- 60, depression score (<17) | Pregnant, lack of Danish language skills, unwillingness to use a phone for examination purposes, severely ill (e.g., schizophrenia spectrum) | Bipolar Disorder | SCAN^4^  interview | MONARCA | Multipurpose application (treatment & tracking) |
| Graham et al. (2020) | 146 | 82%  (18%) | 42 | Compatible smartphone (apple or smartphone), elevated symptoms of anxiety or depression | Acutely suicidal, unappropriated diagnosis, treatment for psychotherapy and if the medication was stable for over 2 weeks | Transdiagnostic | GAD7^10^ & PHQ^5-^8 | IntelliCare | Multipurpose application (treatment & tracking) |
| Hensler et al. (2022) | 179 | 91.6%  (8.4%) | 42.3 | Aged 18+, resident in Sweden with Swedish verbal and written comprehension, has smartphone, traumatic event in past 2 years according to DSM5 and mild to severe symptoms using PTSD check list. | Life threatening or harmful living conditions, current or pending psychotherapy, medical treatment changes and medication with counter medication. | PTSD | DSM-5^6^ | PTSD Coach | Multipurpose application (treatment & tracking) |
| Kauer et al. (2012) | 118 | 63%  (37%) | 17 | Age 14-24, speak proficient English, mild to moderate mental health issue by GP K10^9^(16>) | A psychiatric or medical condition that impedes to have informed consent | Depression | K10^9^ | Mobiletype | Self-monitoring application |
| Kuhn et al. (2017) | 120 | 69%  (31%) | 39 | Age 18 +, English language skills, owning a mobile phone, having been exposed to a traumatic event more than 1 month ago, PCL–C^8^ (>35), and not currently being in PTSD treatment | Did not meet the inclusion criteria | PTSD | PCL–C^8^ | PTSD Coach | Multipurpose application (treatment & tracking) |
| Lewis et al. (2020) | 81 | 30.8%  (69.1%) | 40 | Schizophrenia and related disorders diagnosis, age between 16-65, one or more psychotic episodes in the previous 2 years, including the first psychotic episode | Unable to speak English and/ or unable to give informed consent | Schizophrenia | DSM-5^6^ | ClinTouch | Self-Monitoring application |
| Lüdtke et al. (2018) | 90 | 78%  (22%) | 43 | Need for intervention, age 18-65, using iPhone | Suicidal tendencies | Depression | PHQ^5^-9 | Be Good to Yourself (CBT^19^ third wave) | Treatment mobile application |
| Mantani et al. (2017) | 81 | 55%  (43%) | 41 | Age 25- 59 years, primary major depressive disorder without psychotic features antidepressant-resistant, BDI-2^14^ (<10) after taking one or more antidepressants at an adequate dosage for four or more weeks (stage I, II, or III, not prescribed escitalopram or sertraline, or received CBT^19^ or interpersonal therapy | Did not meet the inclusion criteria | Depression | DSM-5^6^ | Kokoro | Multipurpose (treatment & tracking) |
| Miner et al. (2016) | 49 | 82%  (18%) | 46 | 18 +, English language, not currently receiving treatment for PTSD, having an active e-mail address, PCL–C^8^ (>25) | Did not meet the inclusion criteria | PTSD | PCL–C^8^ | PTSD Coach | Multipurpose application (treatment & tracking) |
| Moberg et al. (2019) | 500 | 74%  (22%) | 30 | Scoring GAD7^10^(5-14) & PHQ^5^- 8 (5-14) | <5 & >14, respectively on GAD7^10^ and PHQ8 | Transdiagnostic | GAD7^10^ & PHQ^5-^8 | Pacifica | Multipurpose application (treatment & tracking) |
| Newman et al. (2020) | 100 | 77%  (23%) | 21.71 | Met diagnostic criteria for GAD | Did not meet diagnostic criteria for GAD | Anxiety | DSM-5^6^ | Mobile application (no name) | Treatment mobile application |
| Nicol et al. (2022) | 17 | 88.2%  (5.9%) | 14.7 | Between 13-17 and had new diagnosis of depression and anxiety in the past 3 months. | Long history of severe depression substance use disorder psychotic illness, OCD, PTSD, panic disorder or specific phobia. Do not have guardian accompanied on visits, did not have access to mobile device for regular use and were unable to read and write English. | Depression and anxiety | DSM-5^6^ | W-GenZ | Multipurpose application (treatment & tracking) |
| O’Toole et al. (2019) | 129 | 44%  (56%) | 29 | Age 18-65, Smartphone for application, symptoms which can indicate interventions period | Severe pathology, substance abuse, inpatient treatment, comorbidity with any other psychopathology apart from mild to moderate depression and anxiety. | Suicidal Behaviour | MDI^11^ & SSF^12^ | LifeApp’tite | Multipurpose application (treatment & tracking) |
| Oh et al. (2020) | 41 | 51%  (49%) | 41 | Age 19 - 60, diagnosis of panic disorder, no changes in medication dosage | Pregnant, neurological illness, comorbid substance use. | Panic Disorder | DSM-5^6^ | Todaki  (Chatbot) | Multipurpose application (treatment & tracking) |
| Possemato et al. (2016) | 20 | 5%  (95%) | 42 | Enrolled in VA primary care, PTSD military symptoms (PCL–C^8^ (>40) | Had treatment in speciality care before study completion, cognitive impairments or suicidal attempt or intent in the previous 2 months, treatment outside of VA primary care or a new or change in dosage of drugs. | PTSD | PCL–C^8^ | PTSD Coach | Multipurpose application (treatment & tracking) |
| Roepke et al. (2015) | 283 | 70%  (30%) | 40 | Age18+, iPhone owner, clinical depression, CES-D^7^ (>16) | Did not meet the inclusion criteria. | Depression | CES-D^7^ | CBT^19^-PPT SuperBetter & General SuperBetter | Treatment mobile application |
| Röhr et al. (2021) | 133 | 38%  (62%) | 33.5 | Syrian refugee residing in Germany, aged 18 to 65 years, experiencing at least one traumatic event and score of 11 to 59) on the Posttraumatic DSM-5, with mobile device | PTSD symptomatology outside inclusion criteria; severe depressive symptoms acute suicidal tendencies current psychotherapy, psychiatric Treatment, and/or psychotropic medication; or pregnancy. | PTSD | DSM-5^6^ | Sanadak | Treatment mobile application |
| Schlosser et al. (2018) | 43 | 62%  (38%) | 24 | Diagnosis of schizophrenia, schizophreniform, or schizoaffective disorder, early course of illness age16- 36 not having substance dependence (6 months prior), clinically stable (1 month prior) ability to provide informed consent, no history of neurological disorders or severe head trauma, English language skills, IQ > 70 | Did not meet the inclusion criteria. | Schizophrenia | DSM-5^6^ | PRIME | Multipurpose application (treatment & tracking) |
| Schwob & Newman (2023) | 82 | 53.6%  (46.4%) | 19.4 | Age 18+ or older, be fluent in English, own an iPhone and meet DSM -5 criteria for social anxiety disorder (SAD) | Excluded if they endorsed mania, psychosis, suicidality, alcohol or substance disorder or any medical or organic disorder that hindered their participation in the study or if currently in psychological or psychiatric treatment for anxiety or any other mental health issues. | SAD | DSM-56 | ImExpsoure | Multipurpose application (treatment & tracking) |
| Steare et al. (2020) | 40 | 30%  (70%) | 29.7 | Aged ≥16 years, had experienced at least one episode of psychosis, were currently on the caseload of an EIP service and owned a Smartphone with an Android operating system. | Lacked capacity to consent to participation, were unable to communicate and understand English, or were considered by their EIP service to pose a high risk to researchers during meetings, even on NHS premises. | Psychosis | ICD-10 | My Journey 3 | Self-Monitoring Application |
| Stolz et al. (2018) | 150 | 65%  (35%) | 35 | Age 18+, own a computer and smartphone with internet; fluent in German; exceeded cut off points for SIAS^16^ and SPS^17^, primary diagnosis of social anxiety disorder | History of psychotic disorder, and medication increase for anxiety and depression in the past month and active suicide plans. | Social Anxiety | DSM-5^6^ | PC and Mobile app (no name) | Treatment mobile application |
| Tighe et al. (2017) | 61 | 63%  (37%) | 25 | Age 18- 35, score PHQ^5^-9 (>10), K10^9^(>25) and had suicidal thoughts in the previous week. | Did not meet the inclusion criteria. | Suicidal Behaviour | PHQ^5^-9 & K10^9^ | ibobbly | Multi-purpose application (treatment and tracking) |
| Vitger et al. (2022) | 194 | 61.9%  (33.5%) | 23.4 | Receiving treatment in OPUS had at least 6 months left of their programme access to a smartphone and understood Danish | Did not meet the inclusion criteria. | Schizophrenia, schizotypal and delusional disorder | N/A | Mobile application (no name) | Multi-purpose application (treatment and monitoring) |
| Acrophobia Questionnaire^1^, Montgomery Asberg Depression Rating Scale^2^, Young Manic Rating Scale^3^, Schedules for Clinical Assessment in Neuropsychiatry^4^, Patient Health Questionnaire^5^, Diagnostic and Statistical Manual of Mental Disorders 5^6^, Centre for Epidemiological Studies Depression questionnaire^7^, PTSD CheckList – Civilian Version^8^, Kessler Psychological Distress Scale^9^, General Anxiety Disorder-7^10^, Major Depression Inventory^11^, Suicide Status Form^12^, International Classification of Diseases^13^, Becks Depression Inventory- 2^14^, Recovery Assessment Scale^15^, Social Interaction Anxiety Scale^16^, Social Phobia Scale^17^ , Behavioural Activation^18^, Cognitive Behavioural Therapy^19^ | | | | | | | | | |

**Supplementary Table 2.** Primary and Secondary Outcomes

| Authors (Publication Year) | Type of mobile application | Clinical effectiveness | Feasibility | Acceptability |
| --- | --- | --- | --- | --- |
| Ben-Zeev et al. (2018) | Multipurpose application (treatment & tracking) | Both conditions improved but no difference. WRAP was more significant in improving recovery (t=2.55, df=289, *p*=.01) and FOCUS in improving quality life scores (t=2.55, df=289, *p*=.001) | FOCUS more likely to commence treatment (90%) and remain fully engaged (56%) compared to WRAP (58% and 40%, respectively). | High satisfaction in both conditions FOCUS (*M*=25.76) and WRAP (*M*=25.56) |
| Bonet et al. (2020) | Self -Monitoring application | After 19 months, ReMindCare had fewer relapses (20% vs 58%) (χ^2^=13.7, P=.001), had fewer visits to urgent care units (χ^2^=7.4, P=.006) and fewer hospitalizations than TAU patients (χ^2^=4.6, P=.03). | ReMindCare group had a compliance rate between 85% and 100%. | Reason of discontinuation included 33% felt suspicious about technology (among these patients, 4 had a relapse while using the app); 40% perceived the app as boring and did not perceive any benefit; and 27% of patients left treatment and did not continue in the program. |
| Bruhns et al. (2023) | Multipurpose application (treatment & tracking) | No significant differences between the groups were found χ²(3) = 1.77;p=.622. | N/A | Slightly positive attitudes towards mobile based intervention. About 86.3% of participants believed that they would feel somewhat better after using the application. More positive side effects i.e. participants felt better using the self-help smartphone app and easier trusting others. |
| Dahne, Collado, et al. (2019) | Multipurpose application (treatment & tracking) | Depressive symptoms compared to TAU χ^2^ = 34.66, df = 1, *p*<0.001; compared to time points χ^2^ = 35.06, df = 14, *p* = 0.001. | Retention rates 72.7% (month 1) and 50% (months 2), post enrolment.  81.8% of used the app ≥8 times, and 36.4% used app ≥56 times. | N/A |
| Dahne, Lejuez, et al. (2019) | Multipurpose application (treatment & tracking) | *M* = –7.51 (3.14), p = .02 (Moodivate vs TAU)  *M* = –7.68 (3.62), p = .03 (MoodKit vs TAU)  Depression symptoms in Moodivate condition *F*(1, 19) = 4.15, *p* = .056  Unique Value (*M*= 6.10) | Retention rate 90% (week 1) 83% (week 2) 67% (week 3-6) 61% (week 7) 50% (week 8).  71% of participants enter self-assessment >18 times. | N/A |
| Depp et al. (2015) | Multipurpose application (treatment & tracking) | Effectiveness at 6 weeks t(223)=−2.2 p=0.031 and 12 weeks *t*(181)=−2.0, *p*=0.042 . Not effective at 24 weeks | Compliance rate (65%) | Satisfaction questionnaire scores: Intervention (*M=* 9); Control (*M*= 10) |
| Donker et al. (2019) | Treatment mobile application | *b*191 = −9.79; *p* < .001; adjusted R^2^ = 0.52. NNT= 1.7. | Intervention retention rates: 59% (post-test) and 49% (follow up); Control retention rates: 91% (post-test and follow up) | System Usability Scale (M =75.35) |
| Faurholt-Jepsen et al. (2021) | Multipurpose application (treatment & tracking) | There was a significant positive association between daily smartphone-based patient-evaluated stress and the CAR (B: 134.14, 95% CI: 1.35; 266.92, p=0.048 (n=33)). significant positive association between patient-evaluated stress measured using the PSS and patient-evaluated stress measured using smartphones (B: 3.33, 95% CI: 2.02; 4.65, p < 0.0001 (n = 33). | N/A | N/A |
| Faurholt-Jepsen et al. (2015) | Multipurpose application (treatment & tracking) | Primary Analysis: B = −0.34, 95% CI −1.14 to 0.47, p= 0.41  Exploratory Analysis unadjusted B = 2.33, 95% CI 0.10–4.56, *p* = 0.040 and the adjusted *B* = 2.57, 95% CI 0.40–4.74, *p* = 0.020 in manic and non-remitting groups. | N/A | N/A |
| Graham et al. (2020) | Multipurpose application (treatment & tracking) | Recovery from depression (OR, 3.25; 95% CI, 1.54-6.86) anxiety (OR 2.17; 95% CI, 1.08-4.36). Sustained at follow up for both depression (slope, 0.01; 95% CI, –0.09 to 0.10; *p*= .92) and anxiety (slope, 0.02; 95% CI, –0.08 to 0.12; *p*= .67) | Usage score (81%) after 8 weeks follow up. | N/A |
| Hensler et al. (2022) | Multipurpose application (treatment & tracking) | Access to PTSD Coach led to a greater decrease in posttraumatic stress after 3 months compared with the waitlist (Cohen d=−0.45, 95% CI −0.70 to −0.20). Access to app show clinically significant improvement (χ21,150=4.62; P=.03) and less likely to fulfil the criteria for probable PTSD than participants on the waitlist after 3 months (χ21,150=7.74; P=.005). However, we detected no difference between conditions in remission from probable PTSD | N/A | Participants with access to PTSD Coach found the app slightly to moderately helpful. sum score on helpfulness was 23.11 (SD 14.32; n=71). Most participants (50/69, 72%) were moderately or very satisfied with the app (n=69, mean 2.22, SD 1.07). |
| Kauer et al. (2012) | Multipurpose mobile application (tracking & predicting) | Increase in emotional awareness χ2 = 11.3, p= .04  Awareness of emotion predicted depressive symptoms κ2=.54 (95% CI .426–.640). | N/A | N/A |
| Kuhn et al. (2017) | Multipurpose application (treatment & tracking) | PTSD symptoms (F(1, 117) = 4.55, p= .035), depression symptoms (F(1, 117) = 7.63, p = .007), and psychosocial functioning (F(1, 117) = 8.34, p=.005). Clinically significant PTSD symptom improvement (*p=*.018) than waitlist participants | *M*=1.29 days of use per week correlated with their self-reported average days used per week (r = .51, p =.01). | N/A |
| Lewis et al. (2020) | Self-Monitoring application | Overall, no differences. However, in London centre found significant reduction in positive symptoms after 12 weeks of ClinTouch-enhanced monitoring in the early psychosis subsample (adjusted mean difference –3.04; CI –5.49, –0.59; P=.016. | 95% stayed in the trial for 12 weeks 84% responding to at least 33% of beep alerts adherence was 60%. Healthcare professionals (care coordinators) used ClinTouch-enhanced management in app in 100% of cases, with average of 24 times per patient. | 90% continued to use it regularly at 3 months. In these patients, adequate adherence was 84%, defined as responding to >33% of item prompts |
| Lüdtke et al. (2018) | Treatment mobile application | Depression score *F*(1;71) = 0.173, *p* = 0.678; self-esteem score *F*(1;71) = 1.464, *p* = 0.230; quality of life score *F*(1;70) = 0.041, *p*= 0.840. Application and TAU increased self-esteem overtime (*p* = 0.274) | N/A | Client Satisfaction Questionnaire 57% |
| Mantani et al. (2017) | Multipurpose application (treatment & tracking) | Kokoro 2.48 points (95% CI 1.23-3.72, P<.001) lower on PHQ-9 and 4.1 points lower on (95% CI 1.5-6.6, P=.002) lower on BDI-2 and 0.76 points (95% CI –0.05 to 1.58, P=.07) lower on side effects.  Mind maps *M*=11.2 | N/A | N/A |
| Miner et al. (2016) | Multipurpose application (treatment & tracking) | Coach reduced PTSD symptoms (t(19) = -2.31, p= .031). 9 participants had clinically significant improvements to the postcondition assessment, compared to 4 in TAU | PTSD Coach usage (*M*=2.65; *SD*= 1.03) weekly and waitlist (*M*=2.50; SD= 0.83) weekly | Satisfaction 83% prefers to learn new tools to cope with their PTSD symptoms. Also, the app was more convenient than the paper condition |
| Moberg et al. (2019) | Multipurpose application (treatment & tracking) | The Pacifica group was lower in depression (-0.59; CI -0.86 to -0.3; *p*<.001) anxiety (-0.43; CI -0.71 to -0.15; *p* = .003), stress (-1.79; CI -2.74 to-0.84; *p*<.001) and higher on self- efficacy (1.55; CI 0.53 to 2.58; *p* = .003) compared to waiting list | Significant attrition rates in Pacifica condition compared to waiting list χ12 (n=500)=7.7;*p*=.006. | N/A |
| Newman et al. (2020) | Treatment mobile application | App group large-effect reductions in all symptom measures during the treatment period. No significant symptom changes across the six-month follow-up period in both conditions. | N/A | N/A |
| Nicol et al. (2022) | Multipurpose application (treatment & tracking) | PHQ-9 scores at 4 weeks decreased by 3.3 units in the intervention group and 2 units in the wait list control group. The percentage of participants achieving remission at both time points seemed to favour the active intervention, at 67% (2/3) and 0% (0/5) at 4 weeks and 50% (1/2) and 20% (1/5) at 12 weeks, respectively. | 70% agreed with the statement “using the app in the treatment of depression seems possible” | 80% agreed or completely agreeing with the statement “I like using the app”; mean usability score 21.4, SD 1.7, possible range 5 to 25 |
| O’Toole et al. (2019) | Multipurpose application (treatment & tracking) | LifeApp’tite decrease in suicide risk end of treatment (F(1, 138.7) = 7.2, *p*= .008, d= 0.46) and 3 months follow up (F(1, 351.1) = 65.0, *p*= .001, d = 0.86) compared to TAU however No between group differences after treatment (*p* = .732, d = 0.05) and follow up (*p* = .467, d = 0.11) | N/A | N/A |
| Oh et al. (2020) | Multipurpose application (treatment & tracking) | Panic disorder symptoms Chatbot versus TAU (t20 = 2.68; *p* = 0.01); reduced phobia (t20 = −2.94; p < 0.01) and helplessness score (t20 = 2.16; p = 0.04) | Retention rate high 80% (SM; n= 8) and 100% (CS; n= 10).  Usage (*M*=9 day for) over 4 weeks.  Usability scores higher in Chatbot vs TAU (64.5 ± 17.0, and 69.5 ± 17.2, respectively; p = 0.35). | N/A |
| Possemato et al. (2016) | Multipurpose application (treatment & tracking) | SM and CS reduced PTSD score (SM= 2.8 (9), *p*=.02; CS= 5.4 (9), *p*≤.01) for social functioning in in CS (−2.0 (9), *p*=.02) | Feasibility was higher than control. Usage is higher in CS than SM over 8 weeks. 5.1 (*SD*= 1.9, range=1–8) PTSD symptoms and 11.7 (*SD*= 6.2, range=4–22) Learn topics, and they utilized 5.3 (*SD*=2.7, range= 3–8) Manage categories. | Higher referral in CS PTSD Coach vs SM PTSD Coach condition (x2(1,18)=7.9 p≤ .01) |
| Roepke et al. (2015) | Treatment mobile application | Depressive symptoms compared to control *t*(276) = - 3.90, *p* < 0.001 | Retention rates were low with 26.15 % (post-test) and 18.34% (follow-up) | N/A |
| Röhr et al. (2021) | Treatment mobile application | ITT no change in DSM-5 scores, but use of app showed low self-stigma after 4 weeks (SSMIS-stereotype agreement: d=0.86, 95% CI 0.46 to 1.25; stereotype application: d=0.60, 95% CI 0.22 to 0.99) and after 4 months (d=0.52, 95% CI 0.12 to 0.92; d=0.50, 95% CI 0.10 to 0.90), the IG showed significantly lower values in self-stigma than the CG. | Total attrition was 12.8% (17/133). usability score of 78.9 | N/A |
| Schlosser et al. (2018) | Multipurpose application (treatment & tracking) | PRIME increasing motivated behaviour (F(1,56) = 4.75, *p* = .03), increasing likelihood of positive future outcomes (F(1,56) = 4.66, *p* = .04). PRIME compared to control had higher decrease of defeatist beliefs F(1,57) = 5.58, *p* = .02, depression (F(1,56) = 7.06, *p* = .01), and self -efficacy (F(1,55) = 5.76, *p* = .02) | PRIME usage 4/7 days.  Completed Challenge rate PRIME (91.47%) compared to (83.58%). Self-monitoring higher in TAU (1.94) versus PRIME (1.74) | Satisfaction rated (*M*=8.21; SD= 1.9) for PRIME. The most popular was directly message coaches (*M* =8.38, *SD*= 2.5), and the least popular was self-monitoring (*M*= 6.33, *SD*= 2.4). |
| Schwob & Newman (2023) | Multipurpose application (treatment & tracking) | There was no significant difference.  between self-monitoring (M =1.09; SD =1.17) and IE (M =1.17; SD = 0.72), β=0.54, SE =0.80, Z =0.68, p =.12 In reported number of social situations engaged in between prompts. However, the reported number of social situations avoided between prompts differed significantly by condition such that self-monitoring (M =1.24; SD =0.56) had more avoided situations on average than imaginal exposure (M =0.92; SD =0.43), β=1.24, SE =0.44, Z =2.82, p=.02. | Calculated compliance rates were 59% for IE (requested thrice daily completion) and 62% for self-monitoring (requested 8 times daily completion), which were not significantly different from each other, β =0.04, SE =0.08, Z =0.50, p=.21. | N/A |
| Steare et al. (2020) | Self-Monitoring Application | No difference in relapse (OR 1.41; 95% CI 0.21 to 9.58), | Participants accessed My Journey 3 on a median of 3.22% of the days it was available to them. Eight participants (40%) used My Journey 3 for longer than 30 min in total. 5 participants used app 5 months after downloading it; 1 participant never used the app after the training session 10 stopped using My Journey 3 within the first 3 months after the training session. | Most service user participants found My Journey 3 to be acceptable, and some participants reported a clear benefit from using it. Barriers affecting use lack of clinician support and concerns around data privacy. A key theme for staff did not have the time to provide regular support to participants with My Journey 3. |
| Stolz et al. (2018) | Treatment mobile application | Superior in all SAD measures (t(119.46)= 5.08, *p*= .01, d=1.07). No difference between App and PC. (t(120.75) =1.71, *p*=.09, d =0.30.). Diagnostic response rates higher in active (NNTPC= 3.33; NNTApp = 6.00) versus TAU. | App higher usage (D=0.14, *p*=.01) versus PC and spread throughout the day | N/A |
| Tighe et al. (2017) | Multipurpose application (treatment & tracking) | ibobbly reduced depressive symptoms (t=2.79; df=56.9; p=0.0072) and distress (t=2.44; df=57.5; p=0.0177) compared to waitlist. No difference in impulsivity (t=−1.82; df=29.1; p=0.0792) | High usage 85% of available data (40/61) completed all the activity. | N/A |
| Vitger et al. (2022) | Multi-purpose application (treatment and self-monitoring) | Statistically significant difference between the intervention and control groups in self-perceived patient activation (mean difference 4.39, 95% CI 0.99-7.79; Cohen d=0.33; P=.01), favouring the intervention group. | N/A | High client satisfaction with mobile application with 44.8% of participants scoring more that 29 out of 32. |

Primary and Secondary Outcomes; Mean (M); Significance level (p); Confidence Interval (CI); Standard Deviation (SD); Patient Health Questionnaire- 9 (PHQ-9); Becks Depression Inventory 2 (BDI- 2).

| **Supplementary Table 3.** Location and Study Duration | | | | |
| --- | --- | --- | --- | --- |
| Authors (Publication Year) | Type of mobile application | App name | Location | Duration of app usage |
| Ben-Zeev et al.  (2018) | Multipurpose application (treatment & tracking) | FOCUS | United States of America | 12 weeks |
| Bonet et al.  (2020) | Self -Monitoring application | ReMindCare App | Spain | 19 months |
| Bruhns et al.  (2023) | Multipurpose application (treatment & tracking) | MCT & More/ COGITO | Germany | 4 weeks |
| Dahne, Collado, et al. (2019) | Multipurpose application (treatment & tracking) | Aptívate! (BA^18^) | United States of America | 8 weeks |
| Dahne, Lejuez, et al.  (2019) | Multipurpose application (treatment & tracking) | Moodivate (BA^18^) | United States of America | 8 weeks |
| Depp et al.  (2015) | Multipurpose application (treatment & tracking) | PRISM | United States of America | 10 weeks |
| Donker et al.  (2019) | Treatment mobile application | ZeroPhobia | The Netherlands | 3 weeks |
| Faurholt-Jepsen et al. (2021) | Multipurpose application (treatment & tracking) | MONARCA | Denmark | 6 months |
| Faurholt-Jepsen et al. (2015) | Multipurpose application (treatment & tracking) | MONARCA | Denmark | 6 months |
| Graham et al.  (2020) | Multipurpose application (treatment & tracking) | IntelliCare | United States of America | 8 weeks |
| Hensler et al.  (2022) | Multipurpose application (treatment & tracking) | PTSD Coach | Sweden | 3 months |
| Kauer et al.  (2012) | Multipurpose mobile application (tracking & predicting) | Mobiletype | Australia | 2-4 weeks |
| Kuhn et al.  (2017) | Multipurpose application (treatment & tracking) | PTSD Coach | United States of America | 3 months |
| Lewis et al.  (2020) | Self-Monitoring application | ClinTouch | United Kingdom | 12 months |
| Lüdtke et al.  (2018) | Treatment mobile application | Be Good to Yourself (CBT^19^ third wave) | Germany | 4 weeks |
| Mantani et al.  (2017) | Multipurpose application (treatment & tracking) | Kokoro | Japan | 9 weeks |
| Miner et al.  (2016) | Multipurpose application (treatment & tracking) | PTSD Coach | United States of America | 1 month |
| Moberg et al.  (2019) | Multipurpose application (treatment & tracking) | Pacifica | United States of America | 1 month |
| Newman et al.  (2020) | Treatment mobile application | Mobile application (no name) | United States of America | 3 months |
| Nicol et al.  (2022) | Multipurpose application (treatment & tracking) | W-GenZ | United States of America | 12 weeks |
| O’Toole et al.  (2019) | Multipurpose application (treatment & tracking) | LifeApp’tite | Denmark | 8 weeks |
| Oh et al.  (2020) | Multipurpose application (treatment & tracking) | Todaki  (Chatbot) | South Korea | 4 weeks |
| Possemato et al.  (2016) | Multipurpose application (treatment & tracking) | PTSD Coach | United States of America | 8 weeks |
| Roepke et al.  (2015) | Treatment mobile application | CBT^19^-PPT SuperBetter & General SuperBetter | United States of America | 1 month |
| Röhr et al.  (2021) | Treatment mobile application | Sanadak | Germany | 4 weeks |
| Schlosser et al.  (2018) | Multipurpose application (treatment & tracking) | PRIME | United States of America, Canada and Australia | 12 weeks |
| Schwob & Newman  (2023) | Multipurpose application (treatment & tracking) | ImExpsoure | United States of America | 7 days |
| Steare et al.  (2020) | Self-Monitoring Application | My Journey 3 | United Kingdom | 12 months |
| Stolz et al.  (2018) | Treatment mobile application | PC and Mobile app (no name) | Switzerland (deduced from ethical approval and author affiliations) | 12 weeks |
| Tighe et al.  (2017) | Multipurpose application (treatment & tracking) | ibobbly | Australia | 6 weeks |
| Vitger et al.  (2022) | Multi-purpose application (treatment and self-monitoring) | Mobile application (no name) | Denmark | 6 months |
| Acrophobia Questionnaire^1^, Montgomery Asberg Depression Rating Scale^2^, Young Manic Rating Scale^3^, Schedules for Clinical Assessment in Neuropsychiatry^4^, Patient Health Questionnaire^5^, Diagnostic and Statistical Manual of Mental Disorders 5^6^, Centre for Epidemiological Studies Depression questionnaire^7^, PTSD CheckList – Civilian Version^8^, Kessler Psychological Distress Scale^9^, General Anxiety Disorder-7^10^, Major Depression Inventory^11^, Suicide Status Form^12^, International Classification of Diseases^13^, Becks Depression Inventory- 2^14^, Recovery Assessment Scale^15^, Social Interaction Anxiety Scale^16^, Social Phobia Scale^17^ , Behavioural Activation^18^, Cognitive Behavioural Therapy^19^ | | | | |
