# Supplementary material for: Smartphone apps for mental health: systematic review of the literature and five recommendations for clinical translation
Source: BMJ Open. 2025 Feb 11;15(2):e093932. doi: 10.1136/bmjopen-2024-093932 (PMC11815452; doi:10.1136/bmjopen-2024-093932)
Supplement: Supplementary file 2 [file bmjopen-2024-093932.reviewer_comments.pdf]

PEER REVIEW HISTORY

BMJ Open publishes all reviews undertaken for accepted manuscripts. Reviewers are asked to complete a checklist review form and are provided with free text boxes to elaborate on their assessment. These free text comments are reproduced below.

ARTICLE DETAILS

Title (Provisional)

Smartphone apps for mental health: systematic review of the literature and five recommendations for clinical translation

Authors

Almuqrin, Aljawharah; Hammoud, Ryan; Terbagou, Ilham; Tognin, Stefania; Mechelli , Andrea

---

VERSION 1 - REVIEW

**Reviewer**	**1**
**Name**	**Cliffe, Bethany**
**Affiliation**	**University of Bath**
**Date**	**21-Oct-2024**
**COI**	**None**

Thank you for the opportunity to review this systematic review of mental health smartphone apps. I appreciate the authors conducting this research and identifying practical recommendations. I have a few thoughts on how to improve this paper, particularly regarding the inclusion of greater detail in the results:

1. The point is made in the introduction (page 4, line 56 – 59) that mental health apps provide access to an extensive population, including those who live in rural areas. It is worth noting however that people in rural areas can struggle with internet access and connectivity so it may not accessible for all. Also, digital literacy/ exclusion are worth considering. Overall, I'd suggest giving a more balanced view as smartphone apps also have several limitations and are certainly not a conclusive solution.

2. It is helpful that examples are given of what is defined as a mental health disorder on page 6 lines 43-47, however it would also be helpful to know what the limits are of how you are defining mental disorder. For example did you include addiction, etc?

3. The search lasting from June 2020 – January 2024 is very long. Please can the authors say why they chose this length of time, and how often the searchers were re-run within this timeframe?

4. Page 7 lines 11-13, this line seems to be a repetition of the previous statement.

5. Did the authors measure any inter-rater reliability or percentage agreement when screening? If not how did they assess reliability of this process.

6. Page 14 line 57 – instead of resulting do the authors mean reducing?

7. Page 14 line 59 – can the authors please provide more detail about the inconsistent results?

8. Page 15 line 8 – I would argue that 60% isn't a particularly high compliance rate. Similarly, page 15 line 42/43 with 50% retention rate. Are the authors able to justify this, or please reword? It might be helpful to state at the beginning what cut-offs you are using to categorise levels of compliance and retention

9. At times the authors define clinical validity as effectiveness, other times as usefulness. Please can this be edited to be consistent with effectiveness

10. Page 18 lines 15-17 and page 20 line 47 – please reference the previous research you refer to

11. It would be helpful to know what countries the studies are from, particularly given the discussion of mental health in developed and developing countries in the introduction. Perhaps this could be included in the study characteristics table

12. Similarly, the study lengths would be useful to know to help readers understand effectiveness and feasibility over time. Particularly as the importance of the study period is mentioned in the discussion on page 17 line 48

13. Overall it feels like the results could benefit from more detail in places to clarify and expand on findings. As an example, there are some things mentioned in the discussion that aren't explicit within the results, like in the discussion it is mentioned that confidentiality was a concern for patients in several studies, it would be helpful to mention this and any other specific concerns in the results. Similarly, in the discussion you talk about effects on quality of life, this also isn't mentioned in the results. I appreciate that there is more information in the table but I think writing some of this into the results is necessary for readers to get the full picture.

14. Attrition is a common problem with smartphone apps for mental health, and I think it would be helpful for the authors to discuss this more in the discussion on page 17 in the first paragraph of the 'is it feasible to use mental health apps' section

15. There are some words missing in places so proof reading throughout would be required

---

**Reviewer**	**2**
**Name**	**Zerhouni, Oulmann**

Affiliation **Université de Rouen**
Date **26-Oct-2024**
COI **None**

This paper assesses the utilization of mobile apps for mental health disorders, emphasizing the aspects of clinical validity, feasibility, and acceptability. The review encompasses 31 studies involving 27 distinct mental health apps, which are classified into three categories: treatment, self-monitoring, and multipurpose applications. The findings suggest that while smartphone apps demonstrate validity and acceptance as tools for mental health, their feasibility diminishes over time. Furthermore, the review identifies issues such as bias, demographic underrepresentation, and the necessity for more comprehensive assessments in the research. The review is well-written and conducted and I really appreciated it and offers meaningful contributions to the field of digital mental health.

The review's assesses apps across several clinical outcomes and incorporates a risk of bias assessment, which adds to the reliability of the conclusions. The review also provides five practical recommendations for improving the clinical translation of mental health apps. These recommendations include ensuring multi-platform compatibility, integrating app data with clinical records, prioritizing privacy, improving user experience, and involving service users during the development process. Most of my remarks are related to improving the discussion.

Nevertheless, the study has some limitations and that there are areas for improvement. The review indicates that the majority of the studies included younger adults, with a paucity of representation from older age groups, particularly individuals over the age of 50. This may limit the generalizability of the findings. While 48.4% of the included studies were deemed to have a low risk of bias, the remaining studies exhibited varying degrees of concern. It would be beneficial for the review to include a more detailed discussion on how these biases may affect the interpretation of the findings. Additionally, although the review indicates that the feasibility of mental health apps decreases over time, it does not thoroughly analyze the factors contributing to this trend. A deeper discussion of issues such as user engagement, app design, and feature utilization could provide valuable insights.

The review's limited exploration of predictive apps is another area that requires further investigation. Although the review primarily focuses on treatment and self-monitoring apps, it is becoming increasingly evident that predictive applications are playing an increasingly significant role in mental health. Consequently, further research is required to gain a deeper understanding of their potential. In order to strengthen its conclusions, the paper would benefit from explicitly discussing how the findings could guide future studies, particularly in relation to long-term app use and more inclusive demographic representation. Additionally, an expanded discussion on data security and privacy would be valuable, given the increasing concerns surrounding digital health technologies.

The review could be enhanced by greater adherence to PRISMA guidelines in a few key areas. Firstly, the search strategy requires greater transparency. PRISMA recommends the provision of a comprehensive search strategy for at least one database, yet the current description is lacking in detail. The inclusion of this information would serve to clarify the comprehensiveness of the search process.

Secondly, the presentation of the study characteristics could be enhanced by including a summary table. Although the narrative description is beneficial, the inclusion of a table would enhance clarity and facilitate a more straightforward comparison of study details, such as sample size, interventions, and outcomes.

The discussion section addresses limitations such as demographic representation and risk of bias, but could be enhanced by a more integrated approach to these aspects and the review's findings. In particular, the risk of bias should be discussed in the context of the results, in accordance with PRISMA guidelines which emphasise the importance of considering bias in the context of study conclusions.

VERSION 1 - AUTHOR RESPONSE

Reviewer: 1
Dr. Bethany Cliffe, University of Bath
Comments to the Author:

Thank you for the opportunity to review this systematic review of mental health smartphone apps. I appreciate the authors conducting this research and identifying practical recommendations. I have a few thoughts on how to improve this paper, particularly regarding the inclusion of greater detail in the results:

1. The point is made in the introduction (page 4, line 56 – 59) that mental health apps provide access to an extensive population, including those who live in rural areas. It is worth noting however that people in rural areas can struggle with internet access and connectivity so it may not accessible for all. Also, digital literacy/ exclusion are worth considering. Overall, I'd suggest giving a more balanced view as smartphone apps also have several limitations and are certainly not a conclusive solution.

Response: Thank you for your helpful feedback. We agree with your perspective on the challenges of internet connectivity and digital literacy. In response, we have added a section to the "Limitations of current smartphone applications" in the Discussion, highlighting that while mental health apps can improve accessibility, unreliable internet connectivity in rural regions and issues of digital literacy may hinder their effectiveness. This addition provides a more balanced view of the potential limitations of mental health apps:

"Despite the many benefits, mental health apps have limitations. Firstly, while it is important to note that a majority of the global population use smartphones(3), most users come from higher-income households(78), limiting access for those with lower socio-economic status. While mental health apps can improve accessibility, particularly for individuals in areas with limited mental health services, their effectiveness may be hindered by barriers such as unreliable internet connectivity in rural regions, which can restrict their functionality and impact(79). Furthermore, digital literacy and the risk of digital exclusion present significant challenges, especially in individuals who are still unfamiliar with smartphones or apps (80)."

2. It is helpful that examples are given of what is defined as a mental health disorder on page 6 lines 43-47, however it would also be helpful to know what the limits are of how you are defining mental disorder. For example did you include addiction, etc?

Response: Thank you for your comment. We appreciate the opportunity to clarify the scope of our inclusion criteria and how mental health disorders were defined in this review. Our inclusion criteria focused on disorders such as depression, anxiety, phobias, obsessive-compulsive disorder, panic disorder, post-traumatic stress disorder, psychosis, bipolar disorder, suicidal ideation/behaviour, and self-harm, as specified in the manuscript. The search terms we included reflected these categories and did not explicitly include addiction or substance use disorders. As a result, studies addressing addiction or related conditions were not within the scope of this review.
We have added a sentence to the "Strengths and weaknesses of the current systematic review" section in the Discussion to highlight this:

"Lastly, the review excluded certain mental health conditions, such as substance use disorders and neurodevelopmental disorders, as these were outside the scope of the current review which focused on common mental health disorders. As a result, the findings may not be generalisable to these populations."

3. The search lasting from June 2020 – January 2024 is very long. Please can the authors say why they chose this length of time, and how often the searchers were re-run within this timeframe?

Response: Thank you for your comment. To clarify, we ran the searches four times between June 2020 and January 2024. This approach allowed us to keep the systematic review up to date, ensuring that newly published studies were included while the manuscript was being prepared for publication. This process ensured the review reflected the most current evidence available on the topic.
We have amended the "Information sources and search strategy" section to clarify:

"This systematic review conducted a comprehensive search that started in June 2020 and ended in January 2024 using PubMed and Ovid database (composed of APA PsycInfo, Global Health, Embase, and Ovid MEDLINE). The searches were run four times

during this period to ensure that the results remained up to date as the review progressed and to incorporate newly published studies relevant to the topic."

4. Page 7 lines 11-13, this line seems to be a repetition of the previous statement.

Response: Thank you for catching the repetition. In response, we have removed the repeated sentence with regards to the databases. The section now reads as follows:

"This systematic review conducted a comprehensive search that started in June 2020 and ended in January 2024 using PubMed and Ovid database (composed of APA PsycInfo, Global Health, Embase, and Ovid MEDLINE). The searches were run four times during this period to ensure that the results remained up to date as the review progressed and to incorporate newly published studies relevant to the topic. Search terms relating to 1) mental health, 2) smartphones and 3) self-management were used (see Appendix A for full search strategy)."

5. Did the authors measure any inter-rater reliability or percentage agreement when screening? If not how did they assess reliability of this process.

Response: Thank you for your comment. While we did not calculate inter-rater reliability or percentage agreement during the screening process, we ensured reliability by having two independent reviewers screen all articles based on the inclusion and exclusion criteria. Any disagreements were resolved by a third reviewer, who evaluated 14 articles where the first two reviewers did not reach a consensus. This approach aligns with PRISMA recommendations for systematic reviews and ensures a rigorous and transparent screening process.
In order to clarify the process, we have made the following changes to the manuscript:

"Two reviewers independently conducted the search and screened articles based on the inclusion and exclusion criteria with a third reviewer resolving inconsistencies. This process ensured the reliability and consistency of study selection. Extracted data included article details (authors, publication year), participant information (sample size, gender, mean age, inclusion/exclusion criteria, diagnosis, diagnostic tool), mental health app information (name, type), and outcome measures (clinical effectiveness, feasibility, acceptability)."

6. Page 14 line 57 – instead of resulting do the authors mean reducing?

Response: Thank you for highlighting the error. We have changed the manuscript to state "reducing" instead of "resulting".

"This systematic review assessed three studies on self-monitoring mental health apps in terms of effectiveness, acceptability, and feasibility. Bonet et al.(45) found that using a self-monitoring app is effective, reducing hospitalizations ($\chi2$=4.6, P=.03), relapses ($\chi2$=13.7, P=.001), and urgent care visits"

7. Page 14 line 59 – can the authors please provide more detail about the inconsistent results?

Response: Thank you for your feedback. We have amended the manuscript with additional details to clarify the 'inconsistent results':

"Bonet et al.(45) found that using a self-monitoring app is effective, reducing hospitalizations ($\chi2=4.6$, $P=.03$), relapses ($\chi2=13.7$, $P=.001$), and urgent care visits ($\chi2=7.4$, $P=.006$)(45), though Steare et al.(59) found no significant impact on clinical outcomes, noting that the trial was not statistically powered to detect effectiveness, and Lewis et al.(60) reported effectiveness primarily in early psychosis, with limited benefits for chronic illness."

8. Page 15 line 8 – I would argue that 60% isn't a particularly high compliance rate. Similarly, page 15 line 42/43 with 50% retention rate. Are the authors able to justify this, or please reword? It might be helpful to state at the beginning what cut-offs you are using to categorise levels of compliance and retention.

Response: Thank you for your comment. While in the current review, we did not define cut-offs used to categorized levels of feasibility and acceptability, we have revised the text to provide a more detailed representation of the feasibility and acceptability findings across the studies. The updated text reads as follows:

"Bonet et al.(45) and Steare et al.(59) reported compliance rates between 85% - 100%, suggesting strong engagements with their respective apps. In Lewis et al.(60), feasibility was reflected by a 95% retention rate over 12 weeks, with 84% of participants achieving acceptable adherence.
Lewis et al.(60) demonstrated high acceptability, with 84% of participants responding to at least 33% of alerts, indicating regular app usage. Similarly, qualitative feedback from Steare et al.(59) suggested that many participants found the app acceptable and reported clear benefits. However, Bonet et al. (2020) observed lower acceptability for participants who suffer from delusions, with 33% expressing suspicion and 40% showing disinterest in the app."

9. At times the authors define clinical validity as effectiveness, other times as usefulness. Please can this be edited to be consistent with effectiveness

Response: Thank you for catching this inconsistency. We have now replaced validity with effectiveness throughout the manuscript to avoid confusion.

10. Page 18 lines 15-17 and page 20 line 47 – please reference the previous research you refer to.

Response: Thank you for the feedback.
Thank you for highlighting the need to clarify the sentence on Page 19 (formerly page 18). Upon review, we found that it repeated information conveyed in the previous two

sentences. We have opted to remove it and replace it with the following:

"Nine studies assessing multipurpose apps found high acceptability, with patients finding them easy to use, convenient, and helpful(43,44,50,62,64,65,72,75). Possemato et al.(43) noted improved satisfaction in those with clinical support. However, differences in app design, target populations, and clinical contexts may influence overall acceptability, highlighting the importance of tailoring apps to user needs(76,77)."

Thank you for catching the missing citation. We have added it to the following sentence: "In previous studies, patients often reported disinterest when using mental health applications, which resulted in missing data and decreased usage(45)." (updated: Page 22)

11. It would be helpful to know what countries the studies are from, particularly given the discussion of mental health in developed and developing countries in the introduction. Perhaps this could be included in the study characteristics table.

Response: Thank you for the insightful feedback. We agree with your suggestion and have incorporated a new table in the supplementary material (Supplementary Table 1), which now provides information about the location of the 31 studies included in the current systematic review.
We have added the following sentence to the "Study Selection and Characteristics" section:

"Further details on the location of each study and duration of app usage can be found in Supplementary Table 1".

We acknowledge this is a limitation in our systematic review, particularly when generalising the findings to developing countries. As we discussed mental health in developing countries in the introduction, we have also addressed this limitation in the discussion and added the following to the "Strengths and weaknesses of the current systematic review" section:

"Furthermore, the geographic distribution of the studies (Supplementary Table 1) also limits the generalisability of the findings. Fourteen of the 31 studies were conducted in the United States, with relatively few studies from low- and middle-income countries. This geographic bias means that the findings may not be fully applicable to populations in developing countries, where mental health apps could be crucial due to a lack of mental health services, resources, and access to care. This underscores the need for further research that includes a greater diversity of locations, particularly in regions where digital health interventions could have a significant impact."

12. Similarly, the study lengths would be useful to know to help readers understand effectiveness and feasibility over time. Particularly as the importance of the study period is mentioned in the discussion on page 17 line 48.

Response: Thank you for your comment. We agree that including study lengths is important for understanding the effectiveness and feasibility over time and have added this information to Supplementary Table 1.

13. Overall it feels like the results could benefit from more detail in places to clarify and expand on findings. As an example, there are some things mentioned in the discussion that aren't explicit within the results, like in the discussion it is mentioned that confidentiality was a concern for patients in several studies, it would be helpful to mention this and any other specific concerns in the results. Similarly, in the discussion you talk about effects on quality of life, this also isn't mentioned in the results. I appreciate that there is more information in the table but I think writing some of this into the results is necessary for readers to get the full picture.

Response: Thank you for your helpful feedback. We agree that adding further detail to the results section provides a more comprehensive overview of the findings. In response, we have now added detail to the results section, specifically addressing confidentiality concerns, quality of life, and other relevant information from the table. We believe this additional information will help readers gain a comprehensive understanding of the findings.

14. Attrition is a common problem with smartphone apps for mental health, and I think it would be helpful for the authors to discuss this more in the discussion on page 17 in the first paragraph of the 'is it feasible to use mental health apps' section.

Response: Thank you for your insightful feedback. We agree that attrition is a common challenge with smartphone apps for mental health and have revised the highlighted paragraph in the discussion to include additional details on the issue. The updated section now reads as follows:

"The feasibility of using mental health apps, defined by an objective measure of usage and retention rates(35), was high for some treatment apps, with higher interaction levels than personal computers(55) and low drop-out rates in certain studies(49). However, attrition remains a common challenge for mental health apps. For example, Roepke et al.(54) reported retention rates as low as 26.15% at post-test and 18.34% during a 6-week follow-up, highlighting the difficulty in sustaining engagement over time. Similarly, Dahne et al.(51) reported retention rates of 90% in the first week and 50% at eight weeks. This decline in feasibility may be due to poor user engagement, repetitive tasks, and privacy concerns. Torous et al.(76) noted that many mental health apps suffer from poor usability, and lack of user-centric design. Such issues can make apps difficult or unenjoyable to use, leading to a loss of interest over time. Aryana et al.(77) emphasised the importance of designing apps that adapt to diverse user contexts and involve user feedback during development. This suggests that while short-term feasibility is promising, long-term retention requires further exploration, potentially through strategies such as enhanced app design and clinical support. More studies are needed to assess long-term feasibility."

15. There are some words missing in places so proof reading throughout would be required

Response: Thank you for the recommendation. We have carefully proofread the manuscript to correct any missing words or errors.

Reviewer: 2
Dr. Oulmann Zerhouni, Université de Rouen

Comments to the Author:
This paper assesses the utilization of mobile apps for mental health disorders, emphasizing the aspects of clinical validity, feasibility, and acceptability.
The review encompasses 31 studies involving 27 distinct mental health apps, which are classified into three categories: treatment, self-monitoring, and multipurpose applications. The findings suggest that while smartphone apps demonstrate validity and acceptance as tools for mental health, their feasibility diminishes over time. Furthermore, the review identifies issues such as bias, demographic underrepresentation, and the necessity for more comprehensive assessments in the research. The review is well-written and conducted and I really appreciated it and offers meaningful contributions to the field of digital mental health.

The review's assesses apps across several clinical outcomes and incorporates a risk of bias assessment, which adds to the reliability of the conclusions.
The review also provides five practical recommendations for improving the clinical translation of mental health apps. These recommendations include ensuring multi-platform compatibility, integrating app data with clinical records, prioritizing privacy, improving user experience, and involving service users during the development process. Most of my remarks are related to improving the discussion.

Response: We thank you for your positive feedback and helpful comments. We have carefully addressed your suggestions in the responses below and incorporated the necessary updates into the revised manuscript.

Nevertheless, the study has some limitations and that there are areas for improvement. The review indicates that the majority of the studies included younger adults, with a paucity of representation from older age groups, particularly individuals over the age of 50. This may limit the generalizability of the findings.

Response: Thank you for your insightful comment. We agree that the underrepresentation of older adults is a significant limitation of the current research. We have updated the discussion section to address this issue, emphasizing the importance of including older adults in future research to ensure the development and validation of mental health apps for diverse age groups.

"However, there are several limitations in the current review. First, the sample was relatively homogenous, mostly middle-aged female participants. Only Kauer et al.(74) included adolescents, and no study included a sample with a mean age of above 50. This underrepresentation of older adults is particularly notable, given the barriers associated with this demographic. Older adults may face additional challenges, such as lower digital literacy and unfamiliarity with smartphone apps(83), which could impact the feasibility and acceptability of these interventions for these groups. Thus, the findings cannot be generalised to a wider population, highlighting an understudied group in digital technologies literature. Future research should prioritise the inclusion of older adults to ensure mental health apps are developed and validated for diverse age groups."

While 48.4% of the included studies were deemed to have a low risk of bias, the remaining studies exhibited varying degrees of concern. It would be beneficial for the review to include a more detailed discussion on how these biases may affect the interpretation of the findings.

Response: We thank you for your comment. We agree that a more detailed discussion on how these biases may affect the interpretation of our findings is important. We have revised the discussion section to expand on this point. It now reads as follows:

"Second, the studies included are of varying quality, with 15 out of 31 studies showing some to high concern in the RoB 2 assessment. The varying levels of bias have important implications for interpreting the findings, as high or unclear risks of bias may overestimate the effectiveness, feasibility, or acceptability of mental health apps. For example, while some high-risk studies, such as Bonet et al.(45), reported high effectiveness with reductions in hospitalisations and relapses, these must be interpreted with caution due to methodological limitations. Issues such as inadequate randomisation, lack of blinding, and selective reporting were observed in several studies, limiting the robustness of their results and emphasising the need for standardized measures. Despite the potential for low-accuracy studies to yield positive results (33,34), the current review prioritised studies with low risk of bias in its conclusions. These limitations highlight the need for future research to adopt robust study designs and transparent reporting to strengthen the evidence base for mental health apps."

Additionally, although the review indicates that the feasibility of mental health apps decreases over time, it does not thoroughly analyze the factors contributing to this trend. A deeper discussion of issues such as user engagement, app design, and feature utilization could provide valuable insights.

Response: Thank you for the helpful feedback. We agree that a deeper discussion of issues such as user engagement, app design, and feature utilisation could provide valuable insights, and have amended the "Is it feasible to use mental health apps?" section to reflect this:

“The feasibility of using mental health apps, defined by an objective measure of usage and retention rates(35), was high for some treatment apps, with higher interaction levels than personal computers(55) and low drop-out rates in certain studies(49). However, attrition remains a common challenge for mental health apps. For example, Roepke et al.(54) reported retention rates as low as 26.15% at post-test and 18.34% during a 6-week follow-up, highlighting the difficulty in sustaining engagement over time. Similarly, Dahne et al.(51) reported retention rates of 90% in the first week and 50% at eight weeks. This decline in feasibility may be due to poor user engagement, repetitive tasks, and privacy concerns. Torous et al.(76) noted that many mental health apps suffer from poor usability, and lack of user-centric design. Such issues can make apps difficult or unenjoyable to use, leading to a loss of interest over time. Aryana et al.(77) emphasised the importance of designing apps that adapt to diverse user contexts and involve user feedback during development. This suggests that while short-term feasibility is promising, long-term retention requires further exploration, potentially through strategies such as enhanced app design and clinical support. More studies are needed to assess long-term feasibility.”

The review's limited exploration of predictive apps is another area that requires further investigation. Although the review primarily focuses on treatment and self-monitoring apps, it is becoming increasingly evident that predictive applications are playing an increasingly significant role in mental health. Consequently, further research is required to gain a deeper understanding of their potential.

Response: Thank you for your feedback. We agree that predictive applications are playing an increasingly important role in mental health. While the current systematic review did not explore the effectiveness, acceptability, or feasibility of single-purpose predictive apps due to a lack of such studies, we did discuss the clinical effectiveness of a predictive feature within a multipurpose app (Kauer et al. 2012). We have added an additional point in the discussion to emphasise the need for more focused research on predictive apps:

“Third, this review did not include single-purpose predict applications due to a lack of such studies. However, one study incorporated a predictive feature within a multipurpose app, demonstrating clinical effectiveness through improved emotional self-awareness and reductions in depressive symptoms(74). This finding highlights the potential of predictive applications in mental health and suggests a need for more focused research to explore their effectiveness, acceptability, and feasibility within digital health interventions.”

In order to strengthen its conclusions, the paper would benefit from explicitly discussing how the findings could guide future studies, particularly in relation to long-term app use and more inclusive demographic representation.

Response: Thank you for the feedback. We agree that discussing long-term app use and more inclusive demographic representation would strengthen the conclusions. We believe that the changes made in previous responses (regarding the demographic

representation of older adults, and utilisation of strategies such as enhanced app design and usability), and the changes outlined below will highlight these points for the readers:

Five recommendations for clinical translations - Point 4:
"4. User experience of apps should be taken into careful consideration: In previous studies, patients often reported disinterest when using mental health applications which resulted in missing data and decreased usage(45). User experience should be an important value during the development of smartphone applications to maximise feasibility and accessibility. Focusing on usability, reducing repetitive tasks incorporating a user-centric design can improve feasibility(76). Focusing on passive data collection and investing in the design of an app can increase its appeal and support long-term retention. Additionally, involving end-users, particularly those from diverse demographic groups who may face challenges with digital literacy, can ensure apps are tailored to meet varying needs."

Conclusion:
"A key aim of using smartphone apps for mental health is to provide tools that can monitor, support treatment, and predict future clinical outcomes. This review found a limited number of validated smartphone apps that have been assessed in terms of clinical effectiveness, feasibility, and acceptability. Overall, smartphone apps are effective and acceptable tools, though feasibility may vary over time, and some studies show bias concerns. As the usage of digital technologies in several fields is quickly evolving, improving effectiveness, feasibility and acceptability is crucial. Future studies should focus on identifying and addressing barriers to long-term feasibility, while emphasising the inclusion of diverse populations. Despite these limitations, smartphone apps offer a cost-effective means to expand resource availability and improve access to care. We hope that this review will assist with the future development of effective, feasible and acceptable smartphone apps for mental health disorders."

Additionally, an expanded discussion on data security and privacy would be valuable, given the increasing concerns surrounding digital health technologies.

Response: Thank you for your feedback. We have made the following changes within our discussion:

"Fourthly, data and privacy concerns present a significant challenge(81,82). Some patients expressed wariness about confidentiality(54,71,75), and in some instances, were uncomfortable responding to self-assessments in a public setting(54). Additionally, data breaches or unauthorised access to sensitive health information could significantly erode trust in mental health apps, a significant barrier to user engagement(76). These concerns can be mitigated by providing transparent privacy policies, adhering to robust encryption standards, and complying with regional data protection laws. Additionally, offering the option to complete assessments at a time when they are in a private setting(44,75) can further address privacy concerns. Addressing these concerns is critical for safeguarding user trust, ensuring confidence, and supporting sustained app usage."

Five recommendations for clinical translations – Point 3:
"3. Privacy and data protection should be a core-value of the app: Several studies highlighted patients' concerns over the confidentiality of their data(54,71,75). Robust encryption and authentication methods to ensure patient confidentially should be implemented during the development of the app, with frequent security audits to maintain trust. Developers should provide transparent, accessible privacy policies and data use statements. All data should be stored in accordance with data protection laws and guidelines."

The review could be enhanced by greater adherence to PRISMA guidelines in a few key areas. Firstly, the search strategy requires greater transparency. PRISMA recommends the provision of a comprehensive search strategy for at least one database, yet the current description is lacking in detail. The inclusion of this information would serve to clarify the comprehensiveness of the search process.

Response: Thank you for your feedback regarding the search strategy. To ensure transparency and adherence to PRISMA guidelines, we have provided the full search strategy with explicit field tags in the appendix and updated the search strategy section in the Methods:

"This systematic review conducted a comprehensive search that started in June 2020 and the final search was conducted on January 14, 2024, using PubMed and Ovid database (composed of APA PsycInfo, Global Health, Embase, and Ovid MEDLINE). The searches were run four times during this period to ensure that the results remained up to date as the review progressed and to incorporate newly published studies relevant to the topic. Search terms relating to 1) mental health, 2) smartphones and 3) self-management were used. Appendix A provides an example of the full search strategy, including applied limits for one of the searches (e.g., randomised controlled trials), while other searches used no additional filters."

Secondly, the presentation of the study characteristics could be enhanced by including a summary table. Although the narrative description is beneficial, the inclusion of a table would enhance clarity and facilitate a more straightforward comparison of study details, such as sample size, interventions, and outcomes.

Response: Thank you for your thoughtful comment. We agree that inclusion of a summary table can enhance clarity and facilitate comparisons across studies. The information requested is presented in the following tables:

- *Table 1. Study Characteristics – which lists the included papers, their sample sizes, mean age, inclusion and exclusion criteria, diagnosis, the diagnostic tool used, the name of the mobile application, and the type of application.*
- *Table 2. Primary and Secondary Outcomes – which lists the papers, the type of mobile application, and the clinical effectiveness, feasibility and acceptability of the apps.*
- *Supplementary Table 1. Location and Study Duration – a newly added table based on reviewer feedback, which provides additional information including the study location and duration of app usage in the listed papers.*

The discussion section addresses limitations such as demographic representation and risk of bias, but could be enhanced by a more integrated approach to these aspects and the review's findings. In particular, the risk of bias should be discussed in the context of the results, in accordance with PRISMA guidelines which emphasise the importance of considering bias in the context of study conclusions.

Response: Thank you for your helpful suggestion. We agree that discussing the risk of bias in the context of the results would enhance the discussion section. We have amended the discussion to provide a more integrated approach:

"Second, the studies included are of varying quality, with 15 out of 31 studies showing some to high concern in the RoB 2 assessment. The varying levels of bias have important implications for interpreting the findings, as high or unclear risks of bias may overestimate the effectiveness, feasibility, or acceptability of mental health apps. For example, while some high-risk studies, such as Bonet et al.(45), reported high effectiveness with reductions in hospitalisations and relapses, these must be interpreted with caution due to methodological limitations. Issues such as inadequate randomisation, lack of blinding, and selective reporting were observed in several studies, limiting the robustness of their results and emphasising the need for standardized measures. Despite the potential for low-accuracy studies to yield positive results (33,34), the current review prioritised studies with low risk of bias in its conclusions. These limitations highlight the need for future research to adopt robust study designs and transparent reporting to strengthen the evidence base for mental health apps."

VERSION 2 - REVIEW

**Reviewer**	**1**
**Name**	**Cliffe, Bethany**
**Affiliation**	**University of Bath**
**Date**	**16-Dec-2024**
**COI**	

Thank you to the authors for taking great care in addressing the points raised previously. I believe this paper is well written, interesting, thorough and I have no further suggestions for the authors. I enjoyed reading this paper and hope to see it published soon.

**Reviewer**	**2**
**Name**	**Zerhouni, Oulmann**

Affiliation **Université de Rouen**

Date **29-Dec-2024**

COI

The authors have satisfacorily answered to my remarks. I have no additionnal point to raise.
